# Supplementary material for: Two C23-Steroids and a New Isocoumarin Metabolite from Mangrove Sediment-Derived Fungus Penicillium sp. SCSIO 41429
Source: Mar Drugs. 2024 Aug 30;22(9):393. doi: 10.3390/md22090393 (PMC11433223; doi:10.3390/md22090393)
Supplement: Supplementary file 1 [file marinedrugs-22-00393-s001.zip › marinedrugs-3168567-supplementary.pdf]

Supplementary Materials

Two C_{23}-Steroids and a New Isocoumarin Metabolite from Mangrove Sediment-Derived Fungus *Penicillium* sp. SCSIO 41429

Lishan Huang [1,†], Chunmei Chen [2,†], Jian Cai [2], Yixin Chen [1], Yongyan Zhu [1], Bin Yang [2], Xuefeng Zhou [2], Yonghong Liu [2], and Huaming Tao [1,*]

1 Guangdong Provincial Key Laboratory of Chinese Medicine Pharmaceutics, School of Traditional Chinese Medicine, Southern Medical University, Guangzhou 510515, China; huanglishan0527@163.com (L.H.); 13676126834@163.com (Y.C.); yongyanzhu0521@163.com (Y.Z.)

2 CAS Key Laboratory Tropical Marine Bio-Resources and Ecology, Guangdong Key Laboratory of Marine Materia Medica, South China Sea Institute of Oceanology, Chinese Academy of Sciences, Guangzhou 510301, China; chenchunmei18@mails.ucas.ac.cn (C.C.); caijian19@mails.ucas.ac.cn (J.C.); yangbin@scsio.ac.cn (B.Y.); xfzhou@scsio.ac.cn (X.Z.); yonghongliu@scsio.ac.cn (Y.L.)

* Correspondence: taohm@smu.edu.cn, Tel.: +86-020-61648770 (H.T.)

† These authors contributed equally to this work.

Contents of Supporting Information

The physicochemical data of the known compounds 3–9 and 11–15. 4
Figure S1. ^{1}H NMR spectrum of cyclocitrinoic acid A (**1**) in DMSO-d_6. 6
Figure S2. ^{13}C NMR spectrum of cyclocitrinoic acid A (**1**) in DMSO-d_6. 6
Figure S3. DEPT 135 spectrum of cyclocitrinoic acid A (**1**) in DMSO-d_6. 7
Figure S4. HSQC spectrum of cyclocitrinoic acid A (**1**) in DMSO-d_6. 7
Figure S5. HMBC spectrum of cyclocitrinoic acid A (**1**) in DMSO-d_6. 8
Figure S6. ^{1}H-^{1}H COSY spectrum of cyclocitrinoic acid A (**1**) in DMSO-d_6. 8
Figure S7. NOESY spectrum of cyclocitrinoic acid A (**1**) in DMSO-d_6. 9
Figure S8. HRESIMS spectrum of cyclocitrinoic acid A (**1**). 10
Figure S9. IR spectrum of cyclocitrinoic acid A (**1**). 10
Figure S10. UV spectrum of cyclocitrinoic acid A (**1**) in MeOH. 11
Figure S11. ECD spectrum of cyclocitrinoic acid A (**1**) in MeOH. 11
Figure S12. ^{1}H NMR spectrum of cyclocitrinoic acid B (**2**) in DMSO-d_6. 12
Figure S13. ^{13}C NMR spectrum of cyclocitrinoic acid B (**2**) in DMSO-d_6. 12
Figure S14. DEPT 135 spectrum of cyclocitrinoic acid B (**2**) in DMSO-d_6. 13
Figure S15. HSQC spectrum of cyclocitrinoic acid B (**2**) in DMSO-d_6. 13
Figure S16. HMBC spectrum of cyclocitrinoic acid B (**2**) in DMSO-d_6. 14
Figure S17. ^{1}H-^{1}H COSY spectrum of cyclocitrinoic acid B (**2**) in DMSO-d_6. 14
Figure S18. NOESY spectrum of cyclocitrinoic acid B (**2**) in DMSO-d_6. 15
Figure S19. HRESIMS spectrum of cyclocitrinoic acid B (**2**). 16
Figure S20. IR spectrum of cyclocitrinoic acid B (**2**). 16
Figure S21. UV spectrum of cyclocitrinoic acid B (**2**) in MeOH. 17
Figure S22. ECD spectrum of cyclocitrinoic acid B (**2**) in MeOH. 17
Figure S23. ^{1}H NMR spectrum of (3*R*,4*S*)-6,8-dihydroxy-3,4,5-trimethyl-7-carboxamidelisocoumarin (**10**) in DMSO-d_6. 18
Figure S24. ^{13}C NMR spectrum of (3*R*,4*S*)-6,8-dihydroxy-3,4,5-trimethyl-7-carboxamidelisocoumari (**10**) in DMSO-d_6. 19
Figure S25. HSQC spectrum of (3*R*,4*S*)-6,8-dihydroxy-3,4,5-trimethyl-7-carboxamidelisocoumari (**10**) in DMSO-d_6. 19
Figure S26. HMBC spectrum of (3*R*,4*S*)-6,8-dihydroxy-3,4,5-trimethyl-7-carboxamidelisocoumari (**10**) in DMSO-d_6. 20
Figure S27. ^{1}H-^{1}H COSY spectrum of (3*R*,4*S*)-6,8-dihydroxy-3,4,5-trimethyl-7-carboxamidelisocoumari (**10**) in DMSO-d_6. 20
Figure S28. NOESY spectrum of (3*R*,4*S*)-6,8-dihydroxy-3,4,5-trimethyl-7-carboxamidelisocoumari (**10**) in DMSO-d_6. 21
Figure S29. HRESIMS spectrum of (3*R*,4*S*)-6,8-dihydroxy-3,4,5-trimethyl-7-carboxamidelisocoumari (**10**). 22
Figure S30. IR spectrum of (3*R*,4*S*)-6,8-dihydroxy-3,4,5-trimethyl-7-carboxamidelisocoumari (**10**). 22
Figure S31. UV spectrum of (3*R*,4*S*)-6,8-dihydroxy-3,4,5-trimethyl-7-carboxamidelisocoumari (**10**) in MeOH. 23
Figure S32. ECD spectrum of (3*R*,4*S*)-6,8-dihydroxy-3,4,5-trimethyl-7-carboxamidelisocoumari (**10**) in MeOH. 23

The strain's (*Penicillium* sp. SCSIO 41429) ITS 1 and 4 sequence of the rDNA. 24
Table S1. Energies at **1** B3LYP/6-311G(d,p) level in methanol. 24
Table S2. Energies at **2** PCM/mPW1PW91/6-311+G(d,p) level in dimethylsulfoxide. 26
Table S3. Energies at **10** B3LYP/6-311G(d,p) level in methanol. 26

The physicochemical data of the known compounds 3–9 and 11–15.

***Cyclo*-(L-Pro-L-Tyr)** (**3**): yellow oil; ^{1}H NMR (500 MHz, DMSO-*d*$_6$) δ_H 7.86 (1H, s, -OH), 7.08 – 7.02 (2H, m, H-2’, 6’), 6.67 – 6.61 (2H, m, H-3’, 5’), 4.24 (1H, t, *J*=5.0 Hz, H-9), 4.09 – 4.00 (1H, m, H-6), 3.40 (2H, dt, *J*=11.7, 8.1 Hz, H$_2$-3), 2.96 – 2.87 (2H, m, H$_2$-10), 2.04 – 1.94 (1H, m, H-5b), 1.75 – 1.68 (2H, m, *J*=6.3, 5.7 Hz, H$_2$-4), 1.45 – 1.34 (1H, m, H-5a); ^{13}C NMR (125 MHz, DMSO-*d*$_6$) δ_C 168.9 (C-7), 165.1 (C-1), 155.9 (C-4’), 130.8 (CH-2’, 6’), 127.0 (C-1’), 114.8 (CH-3’, 5’), 58.4 (CH-9), 56.0 (CH-9), 44.6 (CH$_2$-3), 34.7 (CH$_2$-10), 27.8 (CH$_2$-5), 21.9 (CH$_2$-4).

***Cyclo*-(L-Phe-L-Ala)** (**4**): white powder; ^{1}H NMR (500 MHz, DMSO-*d*$_6$) δ_H 8.10 (1H, s, NH-2), 8.00 (1H, s, NH-5), 7.28 (2H, dd, *J*=8.0, 6.5 Hz, H-10, 14), 7.24 – 7.19 (1H, m, H-12), 7.17 – 7.14 (2H, m, H-11, 13), 4.17 (1H, tt, *J*=3.4, 1.5 Hz, H-6), 3.65 – 3.58 (1H, m, H-3), 3.12 (1H, dd, *J*=13.4, 3.8 Hz, H-8a), 2.86 (1H, dd, *J*=13.4, 4.9 Hz, H-8b), 0.47 (3H, d, *J*=7.0 Hz, H$_3$-7); ^{13}C NMR (125 MHz, DMSO-*d*$_6$) δ_C 167.7 (C-1), 165.8 (C-4), 136.1 (C-9), 130.4 (CH-10, 14), 128.0 (CH-11), 128.0 (CH-13), 126.6 (CH-12), 55.3 (CH-6), 49.7 (CH-3), 38.3 (CH$_2$-8), 19.7 (CH$_3$-7).

Guinolactacin A1 (**5**): orange solid; ^{1}H NMR (500 MHz, DMSO-*d*$_6$) δ_H 8.26 (1H, d, *J*=8.8 Hz, H-8), 8.10 (1H, s, H-2-NH), 7.82 (2H, dd, *J*=5.8, 1.6 Hz, H-5, 6), 7.49 (1H, ddd, *J*=7.9, 5.8, 2.0 Hz, H-7), 4.91 (1H, s, H-3), 3.82 (3H, s, H$_3$-4-Me), 1.59 (1H, m, H-2’), 2.21 – 2.12 (1H, m, H-1’), 1.01 (3H, t, *J*=7.4 Hz, H$_3$-1’-Me), 0.92 – 0.74 (1H, m, H-2’), 0.44 (3H, d, *J*=6.6 Hz, H$_3$-3’);^{13}C NMR (125 MHz, DMSO-*d*$_6$) δ_C 171.6 (C-9), 168.7 (C-1), 164.6 (C-3a), 141.3 (C-4a), 132.6 (CH-6), 128.0 (C-8a), 125.9 (CH-8), 124.4 (CH-7), 117.1 (CH-5), 110.3 (C-9a), 56.9 (CH-3), 36.1 (CH$_3$-4-Me), 36.1 (CH-1’), 20.9 (CH$_2$-2’), 17.6 (CH$_3$-1’-Me), 12.0 (CH$_3$-3’).

N-(N-acetyl-valyl)-phenylalanine (**6**): white powder; ^{1}H NMR (500 MHz, DMSO-*d*$_6$) δ_H 8.10 (2H, t, *J*=9.2 Hz, -NH), 7.28 – 7.22 (4H, m, H-5, 6, 8, 9), 7.19 – 7.14 (1H, t, H-7), 4.69 (1H, td, *J*=9.1, 5.2 Hz, H-2), 4.10 (1H, dd, *J*=8.6, 5.5 Hz, H-11), 2.94 (1H, dd, *J*=13.6, 5.3 Hz, H-3a), 2.73 (1H, dd, *J*=13.6, 9.6 Hz, H-3b), 2.00 (1H, h, *J*=6.8 Hz, H-13), 1.74 (3H, s, H$_3$-16), 0.78 (6H, t, *J*=6.3 Hz, H$_3$-14, 15); ^{13}C NMR (125 MHz, DMSO-*d*$_6$) δ_C 173.1 (C-1), 171.27 (C-10), 169.0 (C-12), 137.9 (C-4), 129.2 (CH-6), 129.2 (CH-8), 128.0 (CH-5), 128.0 (CH-9), 126.2 (CH-7), 57.3 (CH-11), 53.8 (CH-2), 38.3 (CH$_2$-3), 30.2 (CH-13), 22.4 (CH$_3$-16), 19.1 (CH$_3$-14), 17.9 (CH$_3$-15).

Butyrolactone I (**7**): orange solid; ^{1}H NMR (500 MHz, DMSO-*d*$_6$) δ_H 7.51 (2H, d, *J*=8.6 Hz, H-2’, 6’), 6.86 (2H, d, *J*=8.7 Hz, H-3’, 5’), 6.52 (1H, d, *J*=8.1 Hz, H-6”), 6.47 (1H, dd, *J*=8.2, 2.2 Hz, H-5”), 6.37 (1H, d, *J*=2.2 Hz, H-2”), 5.01 (1H, td, *J*=7.2, 3.4 Hz, H-8”), 3.73 (3H, s, H$_3$-5-OCH$_3$), 3.35 (2H, s, H$_2$-6), 2.99 (2H, dd, *J*=7.4, 4.3 Hz, H$_2$-7”), 1.62 (3H, d, *J*=1.6 Hz, H$_3$-10”), 1.53 (3H, d, *J*=1.4 Hz, H$_3$-11”);^{13}C NMR (125 MHz, DMSO-*d*$_6$) δ_C 170.0 (C-5), 168.3 (C-1), 157.7 (C-4’), 153.8 (C-4”), 138.8 (C-2), 131.4 (C-9”), 130.9 (CH-2”), 128.6 (CH-2’, 6’), 128.4 (CH-6”), 126.5 (C-3”), 125.0 (C-3), 123.2 (C-1”), 122.4 (CH-8”), 121.4 (C-1’), 115.7 (CH-3’), 115.7 (CH-5’), 114.1 (CH-5”), 84.7 (C-4), 53.4 (OCH$_3$-C-5), 38.1 (CH$_2$-6), 27.5 (CH$_2$-7”), 25.5 (CH$_3$-10”), 17.5 (CH$_3$-11”).

Penicillenol A1 (**8**): brown oil; ^{1}H NMR (500 MHz, DMSO-*d*$_6$) δ_H 4.09 (1H, qd, *J*=6.4, 2.2 Hz, H-6), 3.82 (1H, s, H-5), 3.53 (1H, s, H-9), 2.95 (3H, s, H$_3$-17), 1.56 (1H, dq, *J*=15.9, 7.1, 5.1 Hz, H-10a), 1.38 (1H, d, *J*=14.4 Hz, H-10b), 1.31 – 1.11 (8H, m, H$_2$-11, 12, 13, 14), 1.07 (3H, d, *J*=6.8 Hz, H$_3$-16), 1.02 (3H, d, *J*=6.4 Hz, H$_3$-7), 0.83 (3H, t, *J*=6.8 Hz, H$_3$-15); ^{13}C NMR (125 MHz, DMSO-*d*$_6$) δ_C 192.4 (C-4), 189.0 (C-8), 173.2 (C-2), 103.4 (C-3), 71.0 (CH-5), 65.7 (CH-6), 35.0 (CH-9), 33.1 (CH$_2$-10), 31.1 (CH$_2$-14), 28.5 (CH$_2$-12), 27.6 (CH$_3$-17), 26.6 (CH$_2$-11), 22.0 (CH$_2$-13), 17.0 (CH$_3$-16), 17.0 (CH$_3$-7), 13.9 (CH$_3$-15).

Penicillenol A2 (**9**): brown oil; ^{1}H NMR (500 MHz, DMSO-d_6) δ_H 4.02 (1H, qd, *J*=6.7, 3.0 Hz, H-6), 3.70 (1H, d, *J*=3.3 Hz, H-5), 3.53 (1H, q, *J*=7.1 Hz, H-9), 2.98 (3H, s, H_3-17), 1.64 – 1.51 (1H, m, H-10a), 1.39 (1H, h, *J*=6.4, 5.2 Hz, H-10b), 1.25 – 1.19 (11H, m, H_2-11, 12, 13, 14; H_3-16), 1.06 (3H, d, *J*=6.8 Hz, H_3-7), 0.84 (3H, t, *J*=6.8 Hz, H_3-15); ^{13}C NMR (125 MHz, DMSO-d_6) δ_C 193.5 (C-4), 189.2 (C-8), 175.1 (C-2), 101.3 (C-3), 66.4 (CH-5), 66.4 (CH-6), 35.2 (CH-9), 33.1 (CH_2-10), 31.1 (CH_2-14), 29.1 (CH_2-12), 28.5 (CH_3-17), 26.6 (CH_2-11), 22.0 (CH_2-13), 16.9 (CH_3-7), 16.9 (CH_3-16), 13.9 (CH_3-15).
Stoloniferol B (**11**): yellow oil; (*c* 0.1, MeOH); ^{1}H NMR (500 MHz, DMSO-d_6) δ_H 11.26 (1H, s, -OH), 6.29 (1H, s, H-7), 4.68 (1H, q, *J*=6.6 Hz, H-3), 3.06 (1H, q, *J*=7.1 Hz, H-4), 1.99 (3H, s, H_3-13), 1.20 (6H, dd, *J*=14.0, 6.8 Hz, H_3-11, 12); ^{13}C NMR (125 MHz, DMSO-d_6) δ_C 168.2 (C-1), 163.4 (C-6), 161.4 (C-8), 142.8 (C-10), 113.9 (C-5), 100.2 (CH-7), 98.1 (C-9), 79.5 (CH-3), 33.7 (CH-4), 19.5 (CH_3-12), 19.4 (CH_3-11), 9.8 (CH_3-13).
Decarboxydihydrocitrinin (**12**): brown oil; ^{1}H NMR (500 MHz, DMSO-d_6) δ_H 6.22 (1H, s, H-7), 4.48 – 4.32 (2H, m, H_2-1), 3.76 (1H, qd, *J*=6.5, 2.5 Hz, H-3), 2.53 (1H, dd, *J*=8.3, 5.7 Hz, H-4), 1.93 (3H, s, H_3-5-Me), 1.11 (3H, s,H_3-3-Me), 1.10 (3H, s,H_3-4-Me); ^{13}C NMR (125 MHz, DMSO-d_6) δ_C 154.1 (C-6), 150.7 (C-8), 137.2 (C-4a), 111.7 (C-5), 110.7 (C-8a), 99.6 (CH-7), 73.4 (CH-3), 58.9 (CH_2-1), 34.6 (CH-4), 20.4 (CH_3-3-Me), 17.9 (CH_3-4-Me), 10.1 (CH_3-5-Me).
Phenol A (**13**): brown oil; ^{1}H NMR (500 MHz, DMSO-d_6) δ_H 6.14 (1H, d, *J*=2.3 Hz, H-4), 6.12 (1H, d, *J*=2.4 Hz, H-6), 3.68 (1H, p, *J*=6.1 Hz, H-8), 2.96 – 2.88 (1H, m, H-7), 1.97 (3H, s, H_3-10), 1.04 (3H, d, *J*=7.0 Hz, H_3-11), 0.95 (3H, d, *J*=6.2 Hz, H_3-9); ^{13}C NMR (125 MHz, DMSO-d_6) δ_C 155.5 (C-1), 155.1 (C-5), 144.5 (C-3), 112.6 (C-2), 104.7 (CH-4), 99.9 (CH-6), 69.2 (CH-8), 41.3 (CH-7), 19.3 (CH_3-9), 15.4 (CH_3-11), 10.6 (CH_3-10).
4-Hydroxy-3,5,6-trimethyl-2*H*-pyran-2-one (**14**): white solid; ^{1}H NMR (500 MHz, DMSO-d_6) δ_H 2.12 (3H, s, H_3-9), 1.84 (3H, s, H_3-8), 1.80 (3H, s, H_3-7); ^{13}C NMR (125 MHz, DMSO-d_6) δ_C 165.9 (C-4), 164.6 (C-2), 154.2 (C-6), 107.1 (C-5), 96.4 (C-3), 16.9 (CH_3-9), 10.2 (CH_3-8), 9.3 (CH_3-7).
4-Methyl-5,6-dihydropyren-2-one (**15**): white oil; ^{1}H NMR (500 MHz, DMSO-d_6) δ_H 5.73 (1H, t, *J*=1.5 Hz, H-3), 4.29 (2H, t, *J*=6.3 Hz, H_2-5), 2.37 (2H, t, *J*=6.3 Hz, H_2-6), 1.96 (3H, d, *J*=1.5 Hz, H_3-7); ^{13}C NMR (125 MHz, DMSO-d_6) δ_C 164.0 (C-2), 159.6 (CH-3), 115.5 (CH-4), 65.7 (CH_2-5), 28.5 (CH_2-6), 22.5 (CH_3-7).

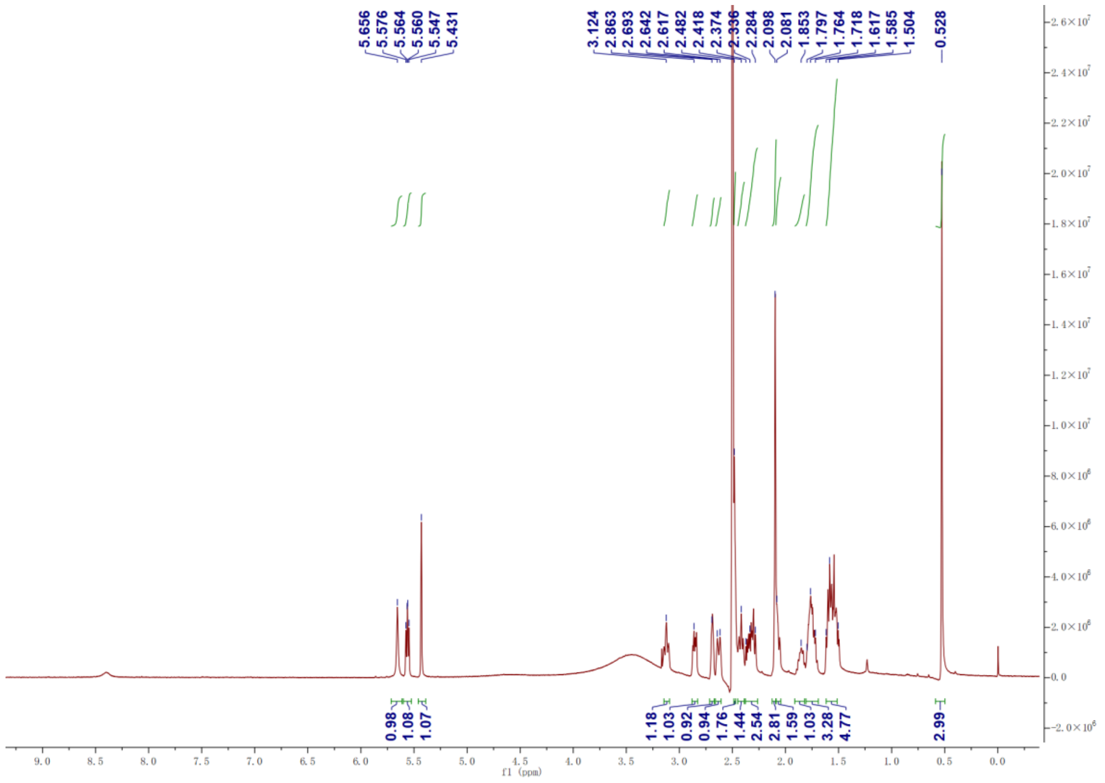

Figure S1. ^{1}H NMR spectrum of cyclocitrinoic acid A (**1**) in DMSO-d_6.

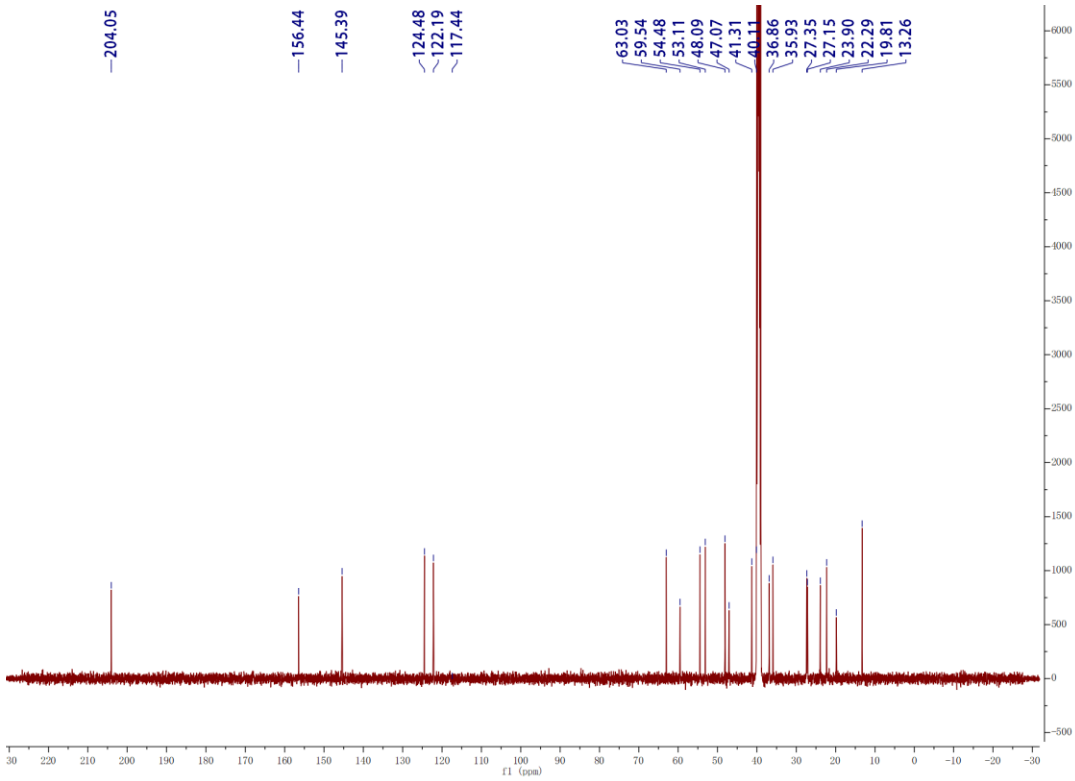

Figure S2. ^{13}C NMR spectrum of cyclocitrinoic acid A (**1**) in DMSO-d_6.

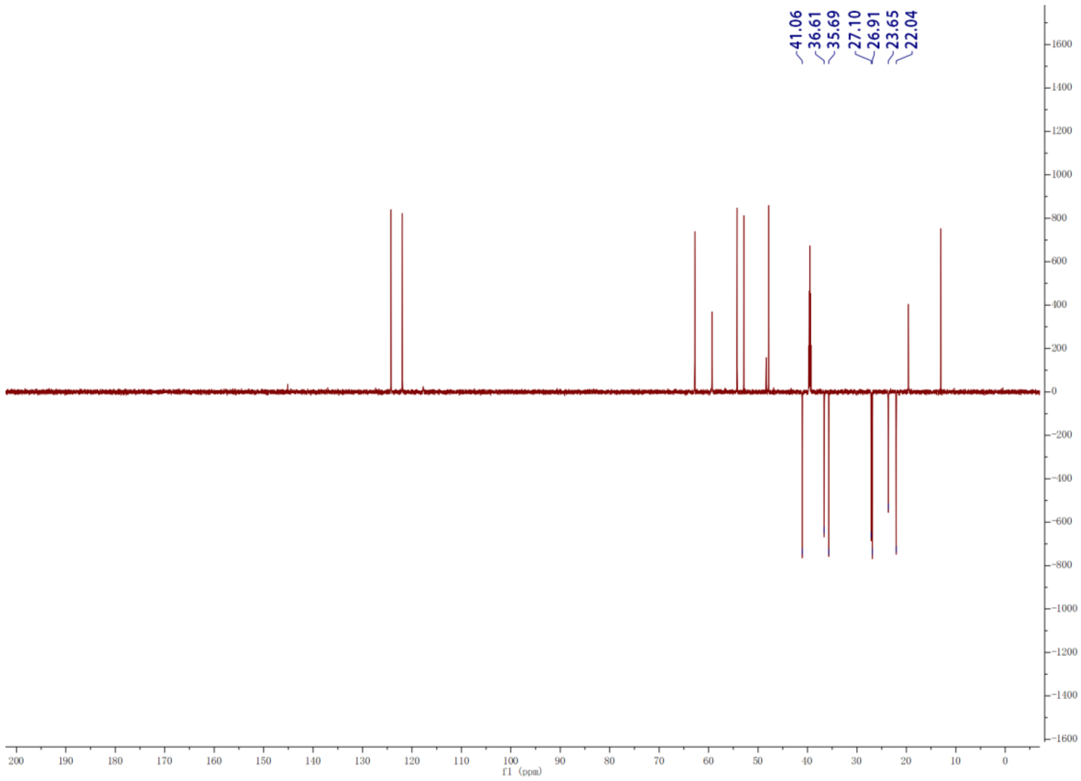

Figure S3. DEPT 135 spectrum of cyclocitrinoic acid A (**1**) in DMSO-d_6.

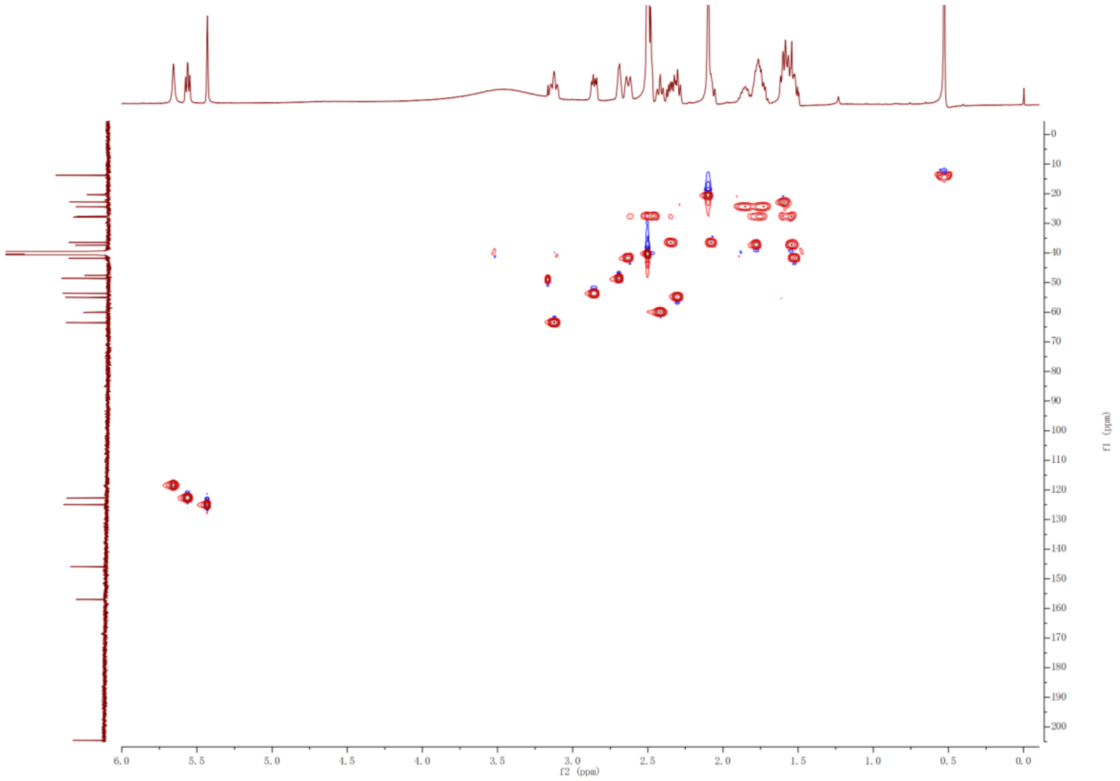

Figure S4. HSQC spectrum of cyclocitrinoic acid A (**1**) in DMSO-d_6.

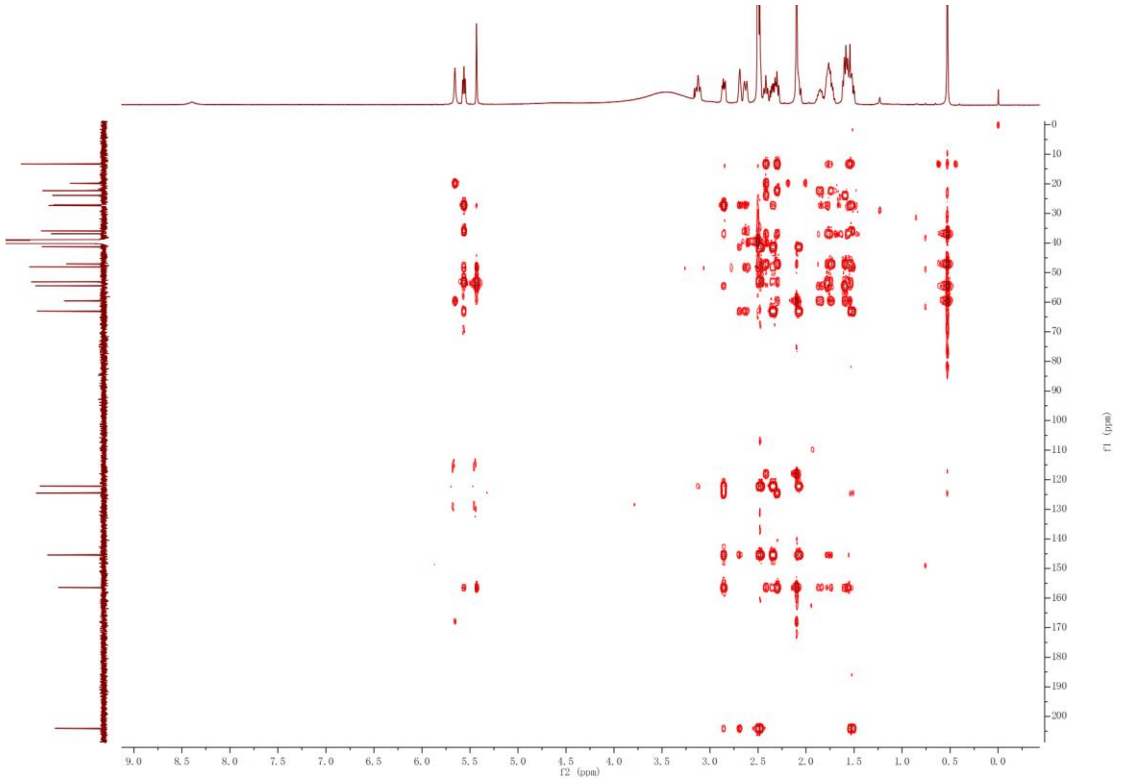

Figure S5. HMBC spectrum of cyclocitrinoic acid A (**1**) in DMSO-d_6.

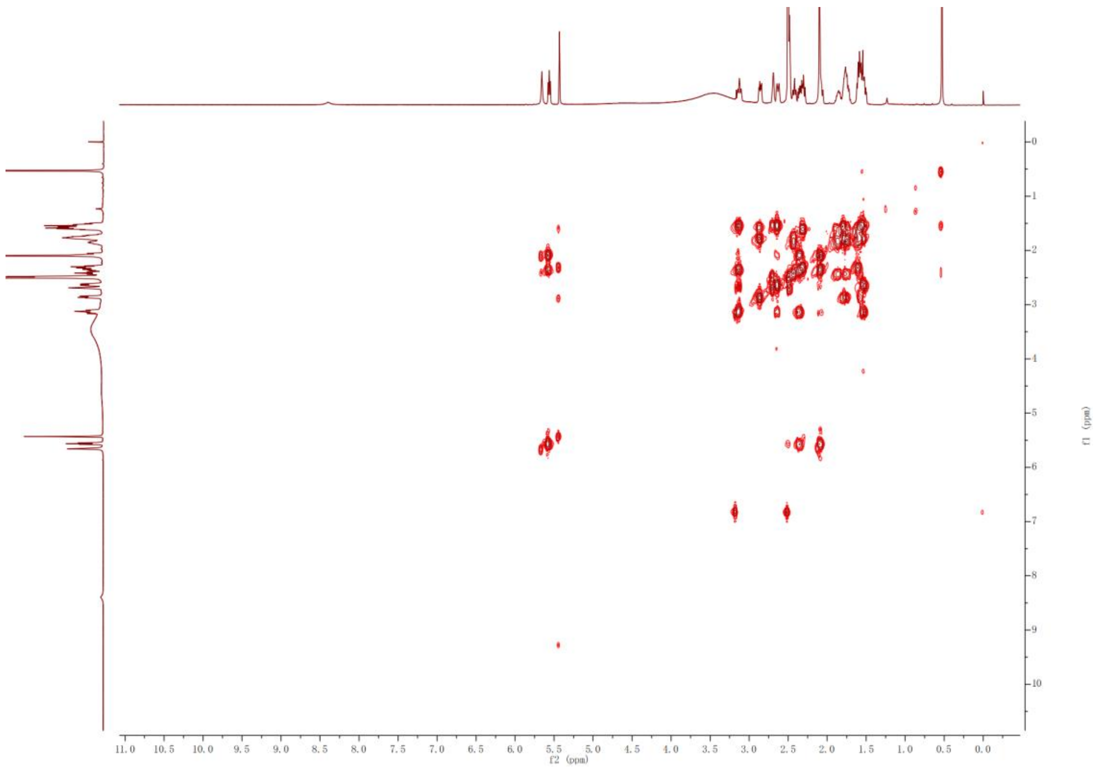

Figure S6. ^{1}H-^{1}H COSY spectrum of cyclocitrinoic acid A (**1**) in DMSO-d_6.

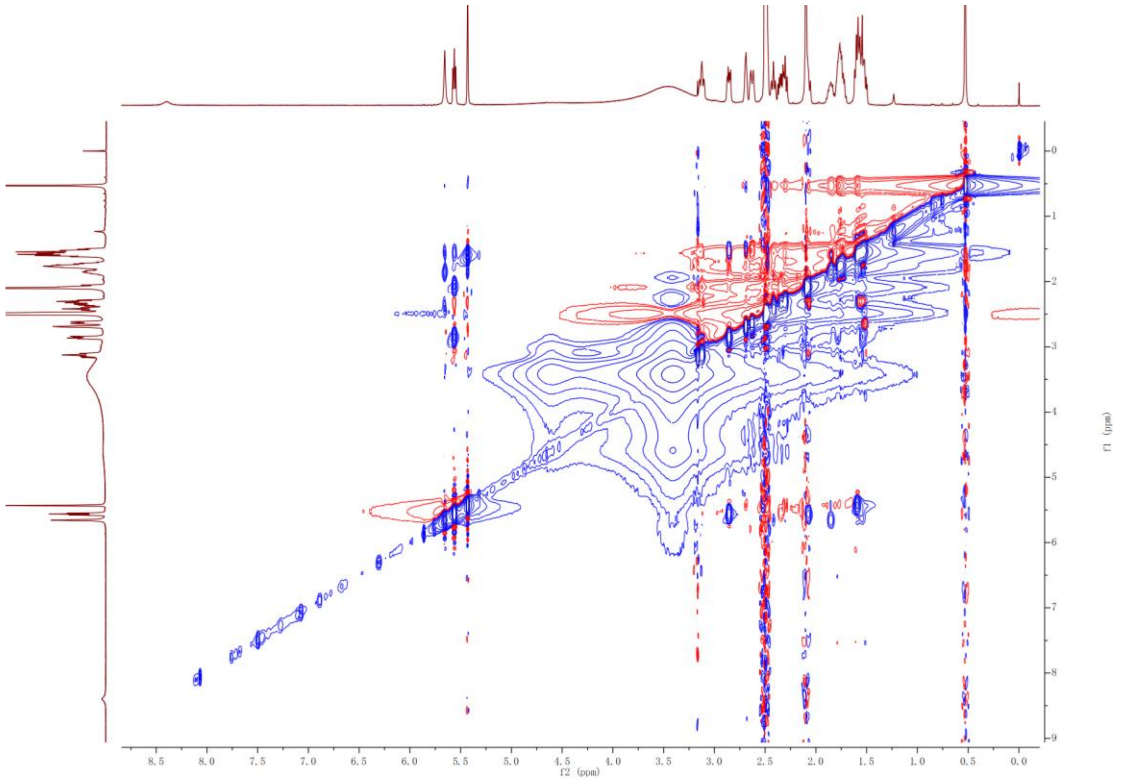

Figure S7. NOESY spectrum of cyclocitrinoic acid A (**1**) in DMSO-d_6.

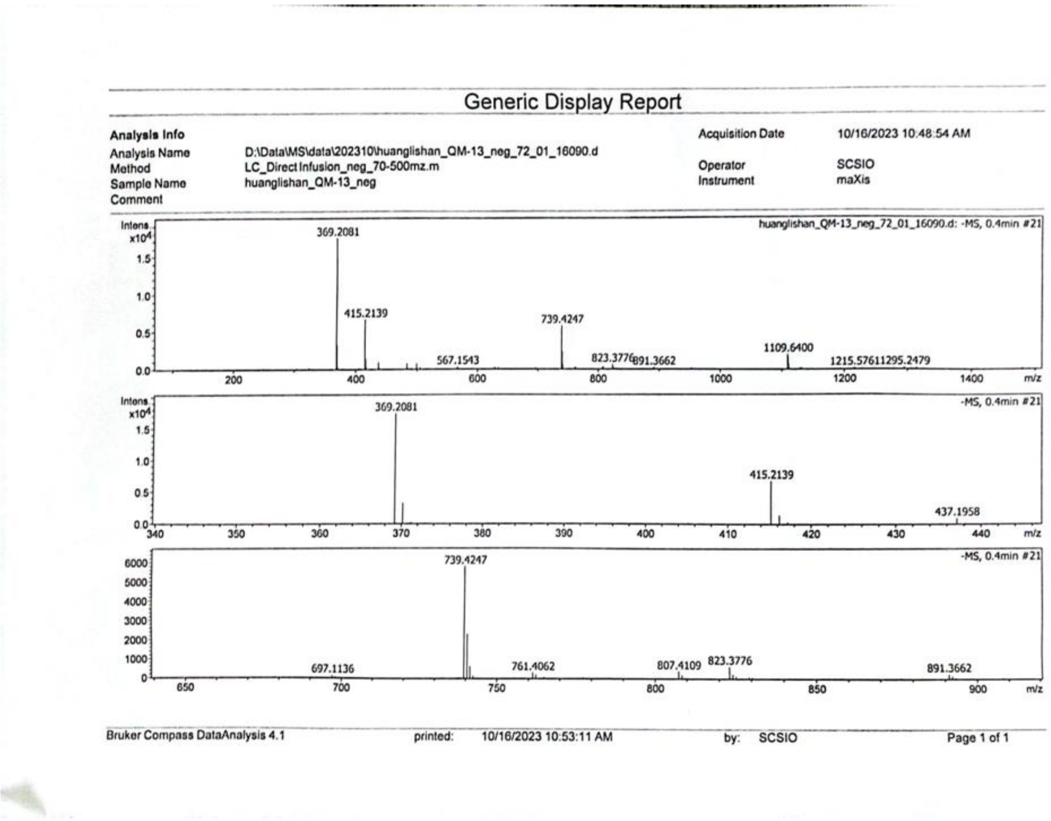

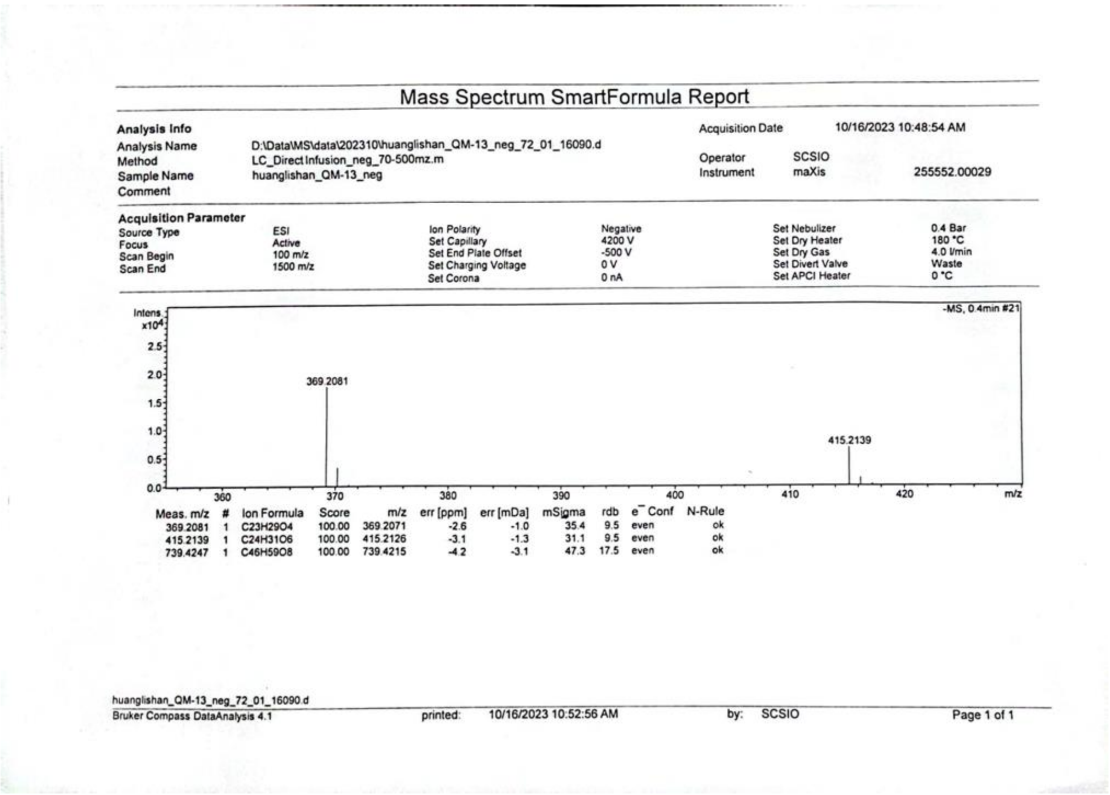

Figure S8. HRESIMS spectrum of cyclocitrinoic acid A (**1**).

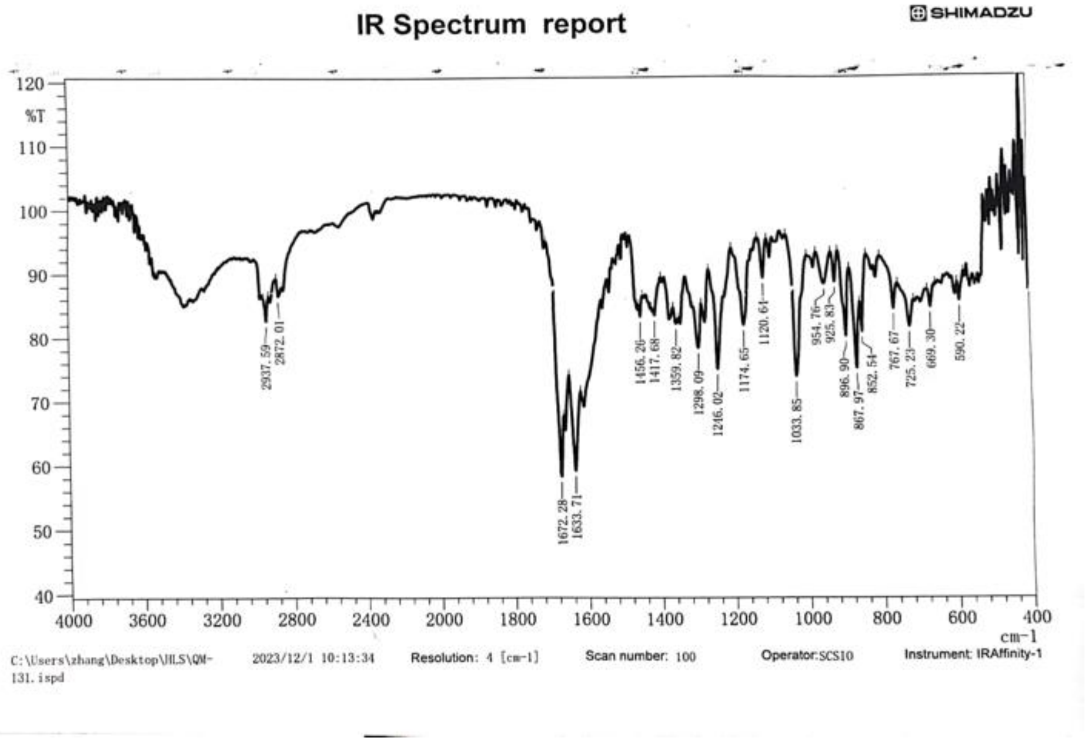

Figure S9. IR spectrum of cyclocitrinoic acid A (**1**).

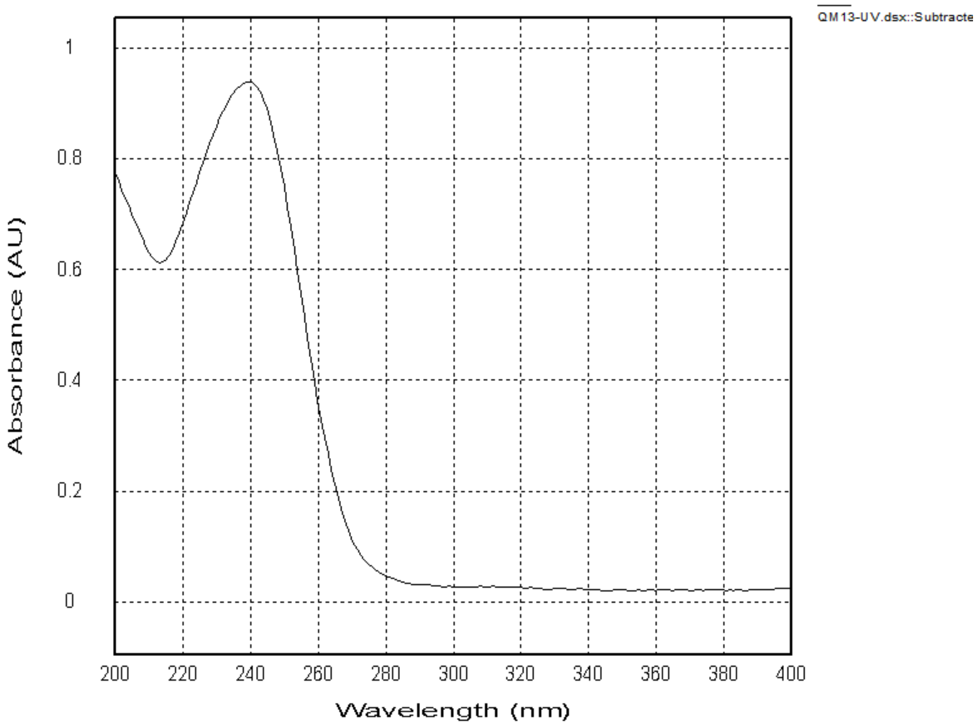

Figure S10. UV spectrum of cyclocitrinoic acid A (**1**) in MeOH.

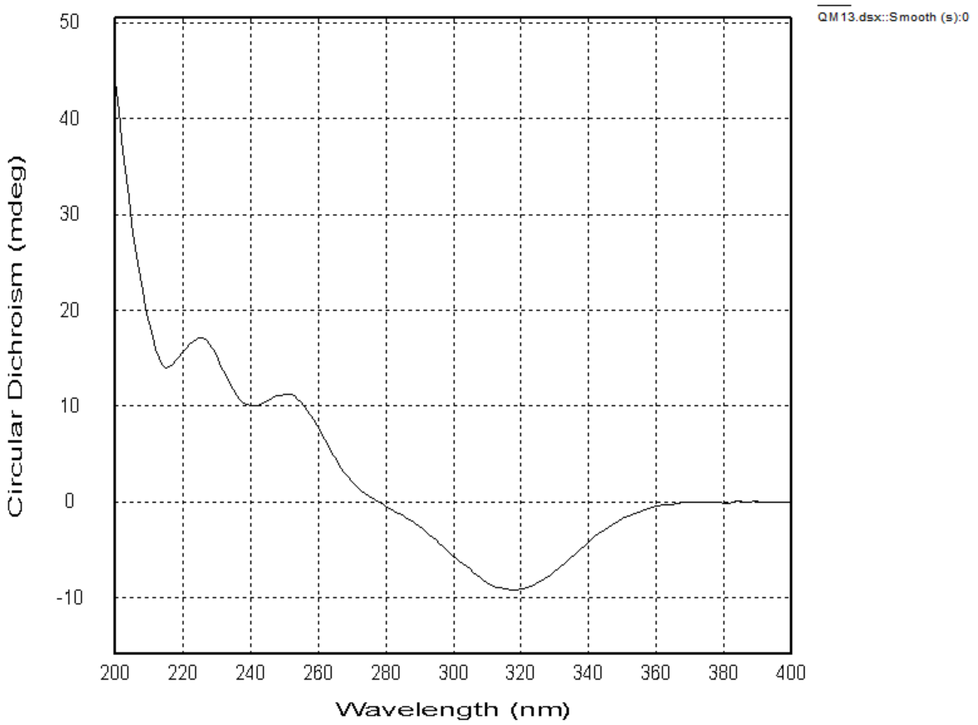

Figure S11. ECD spectrum of cyclocitrinoic acid A (**1**) in MeOH.

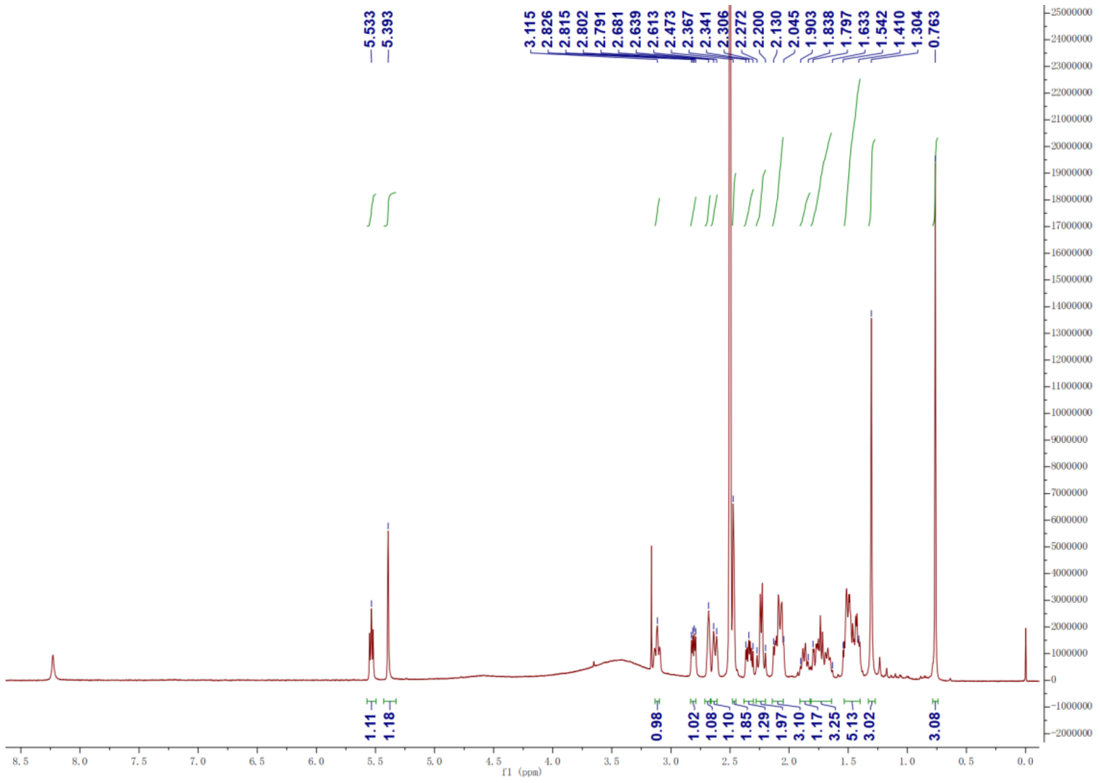

Figure S12. ^{1}H NMR spectrum of cyclocitrinoic acid B (**2**) in DMSO-d_6.

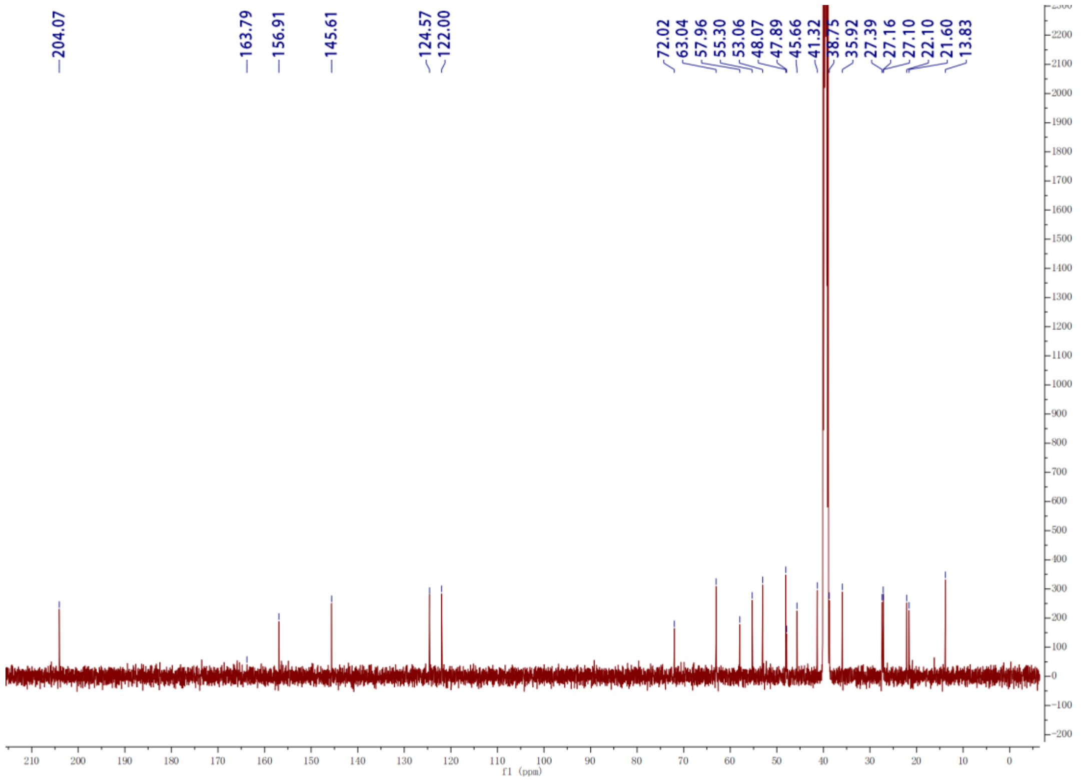

Figure S13. ^{13}C NMR spectrum of cyclocitrinoic acid B (**2**) in DMSO-d_6.

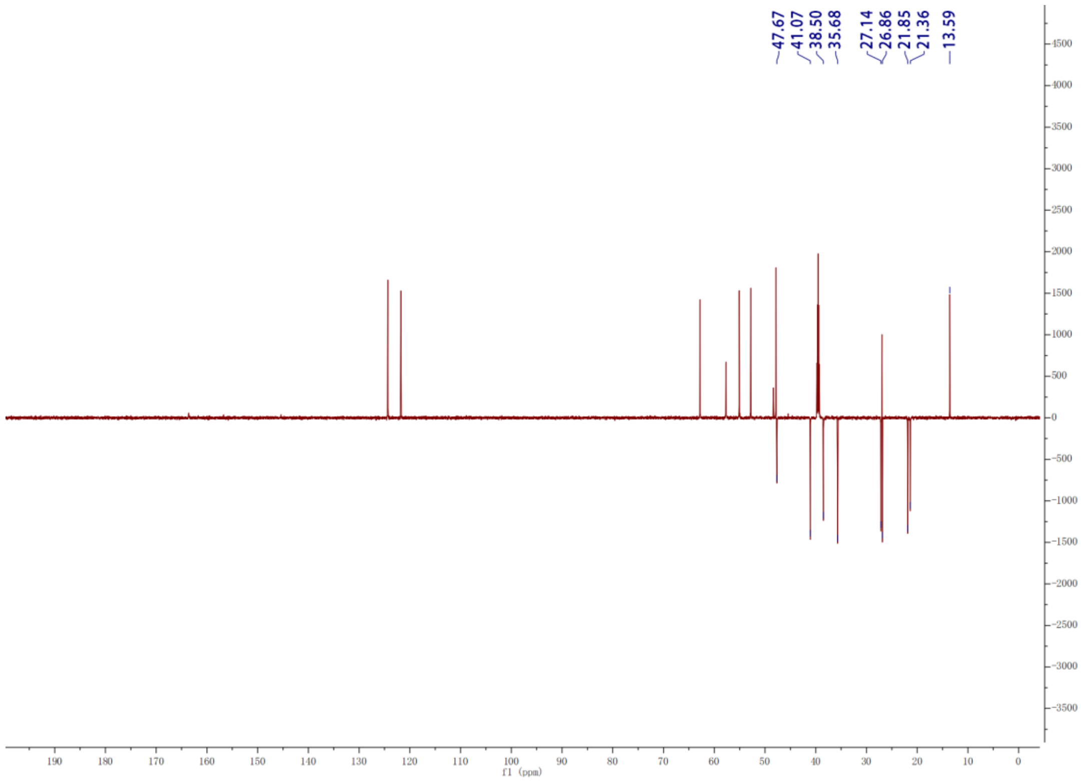

Figure S14. DEPT 135 spectrum of cyclocitrinoic acid B (**2**) in DMSO-d_6.

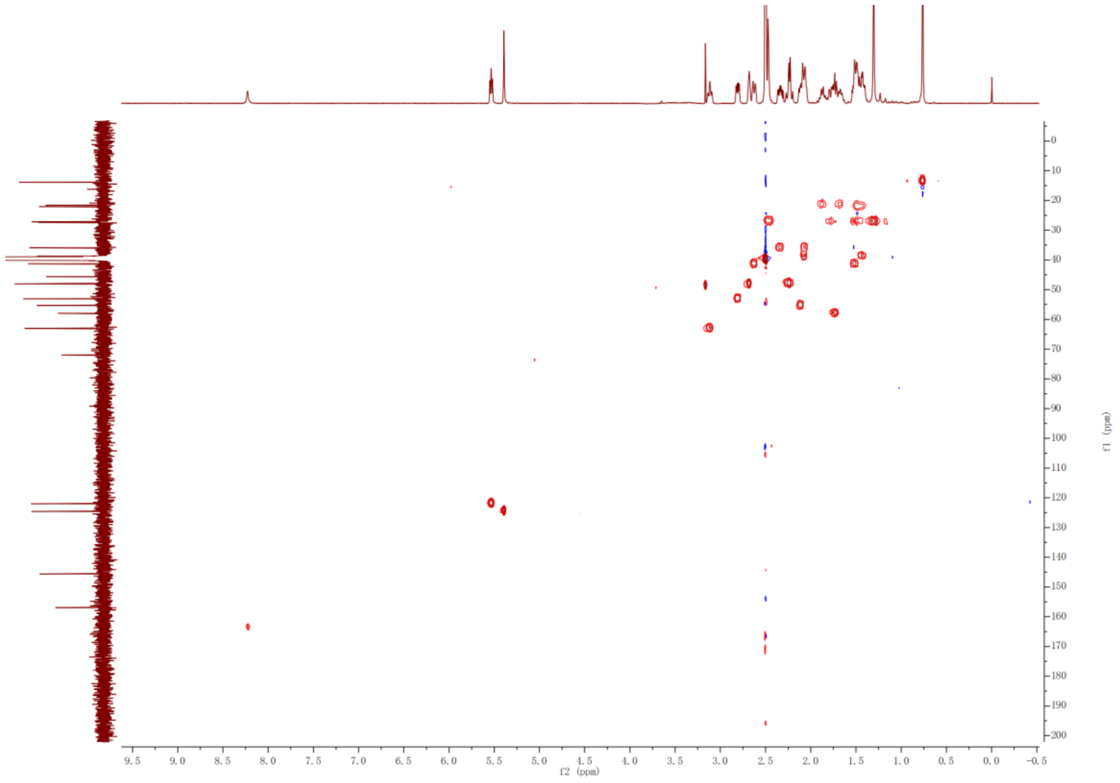

Figure S15. HSQC spectrum of cyclocitrinoic acid B (**2**) in DMSO-d_6.

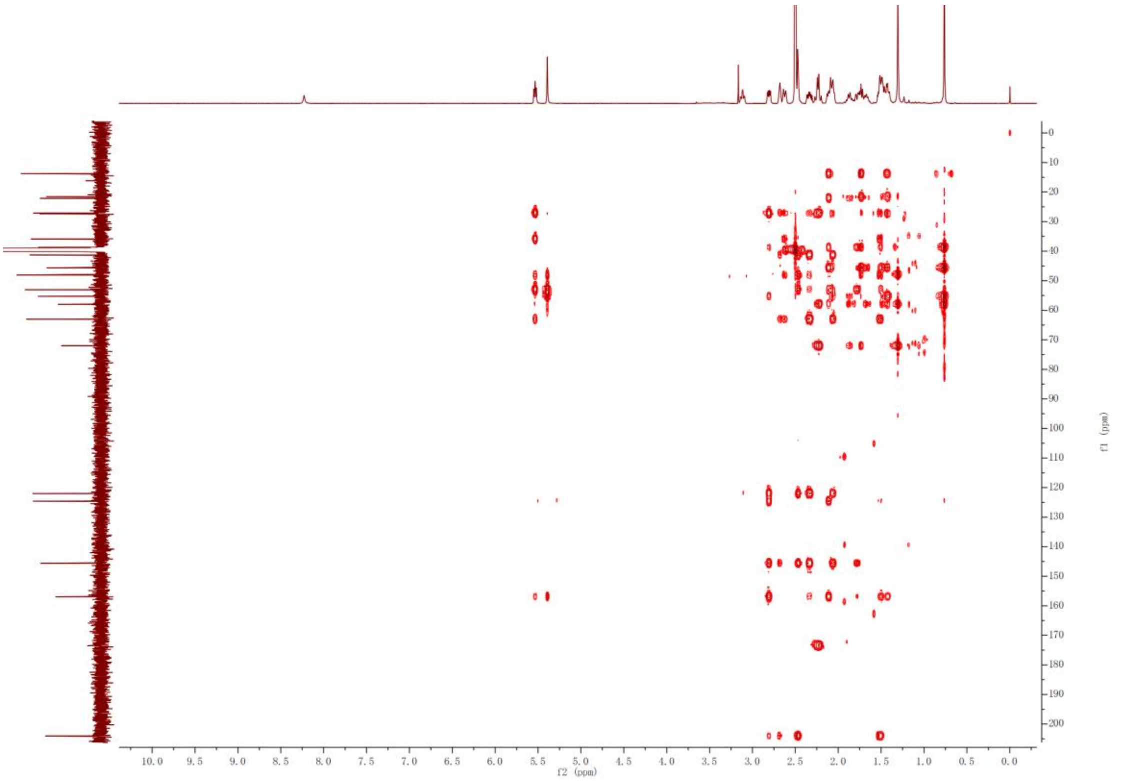

Figure S16. HMBC spectrum of cyclocitrinoic acid B (**2**) in DMSO-d_6.

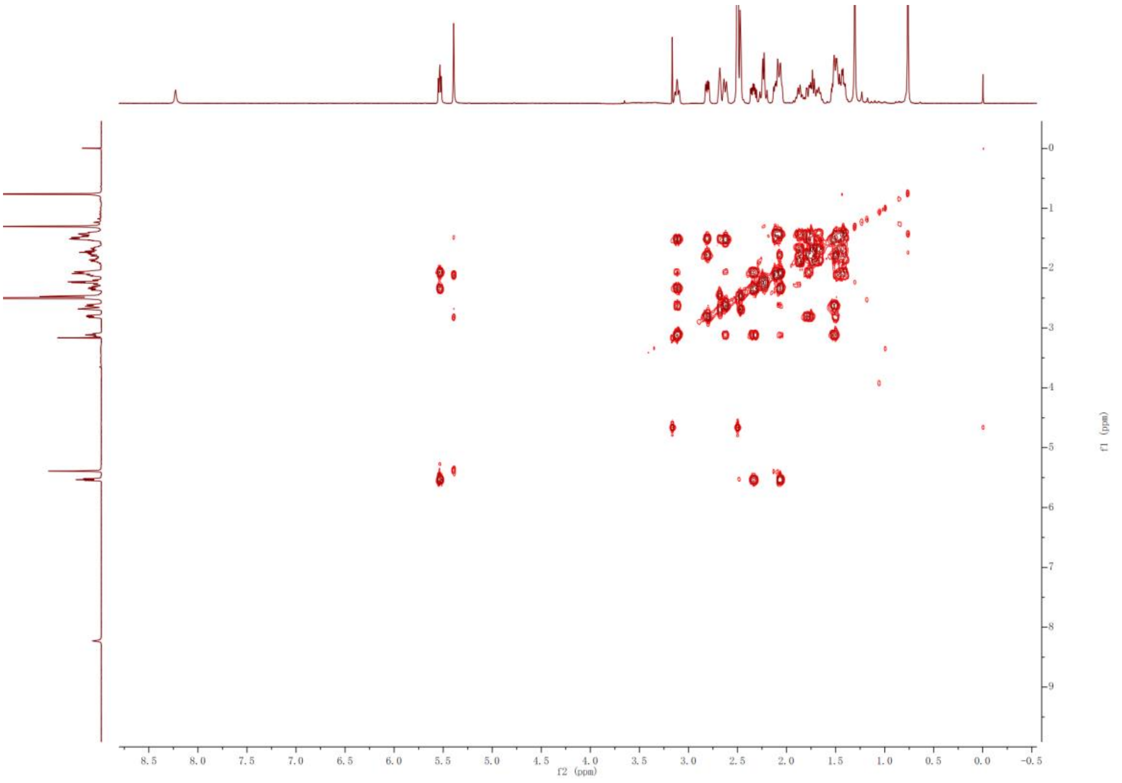

Figure S17. ^{1}H-^{1}H COSY spectrum of cyclocitrinoic acid B (**2**) in DMSO-d_6.

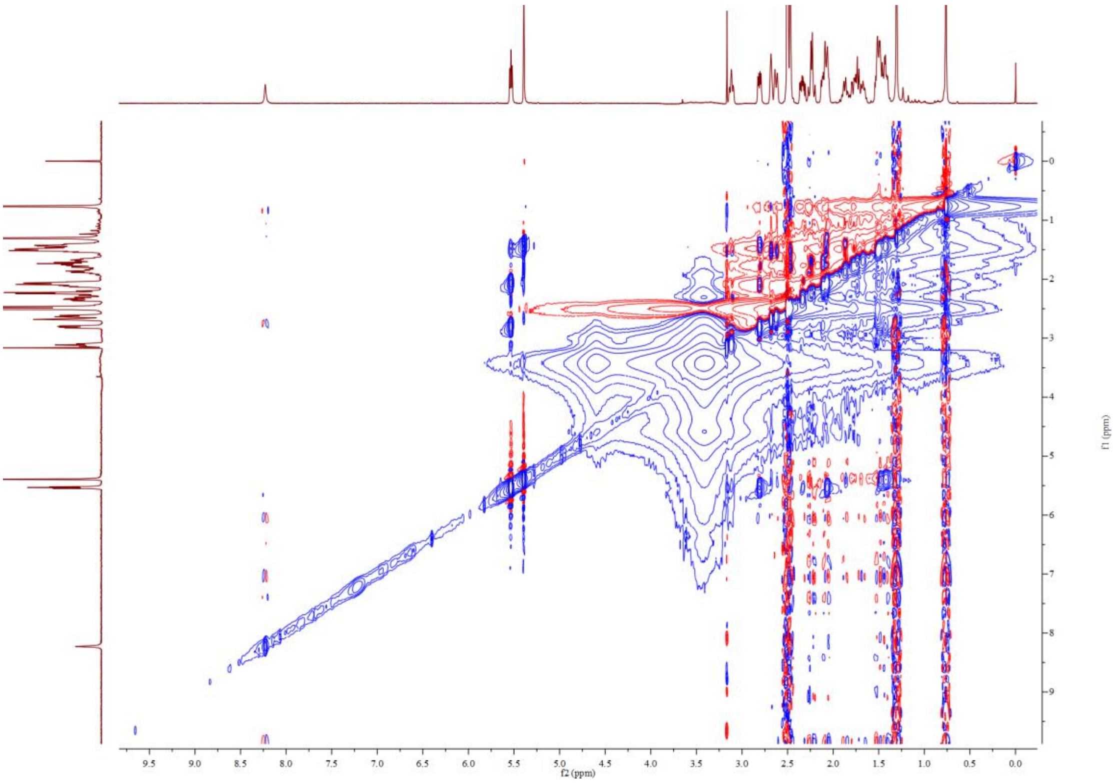

Figure S18. NOESY spectrum of cyclocitrinoic acid B (**2**) in DMSO-d_6.

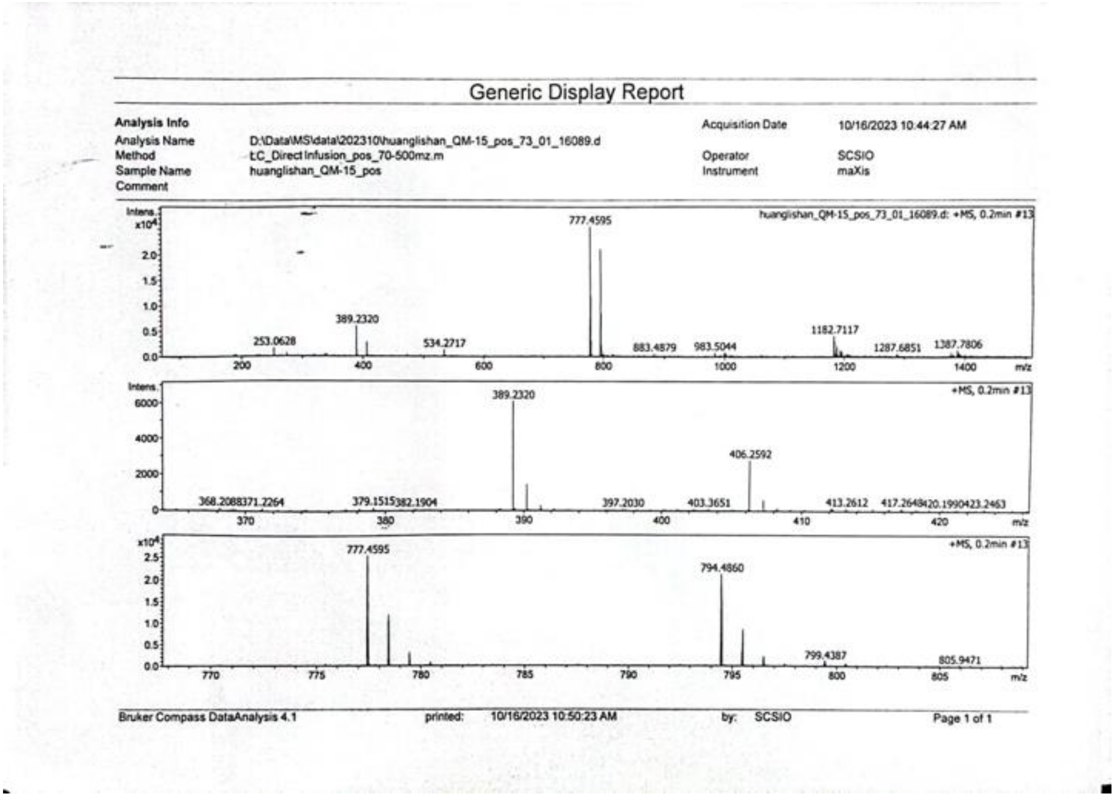

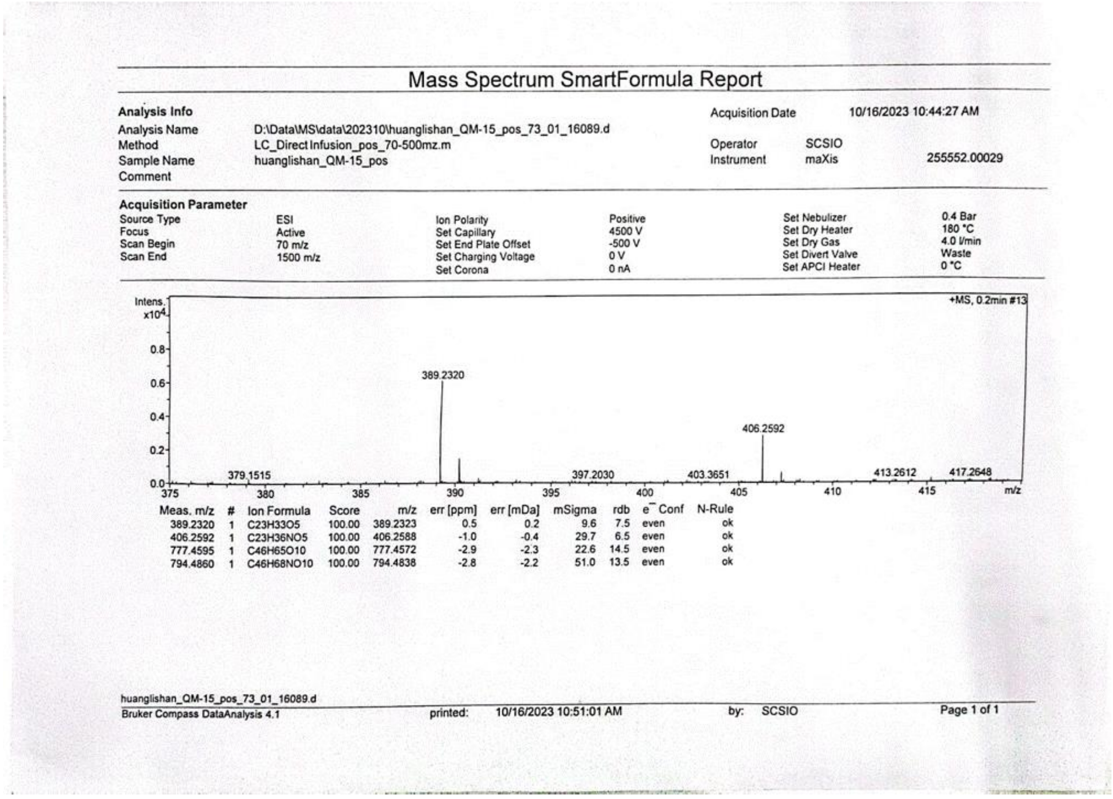

Meas. m/z	#	Ion Formula	Score	m/z	err [ppm]	err [mDa]	mSigma	rdb	e⁻ Conf	N-Rule
389.2320	1	C23H33O5	100.00	389.2323	0.5	0.2	9.6	7.5	even	ok
406.2592	1	C23H36NO5	100.00	406.2588	-1.0	-0.4	29.7	6.5	even	ok
777.4595	1	C46H65O10	100.00	777.4572	-2.9	-2.3	22.6	14.5	even	ok
794.4860	1	C46H68NO10	100.00	794.4838	-2.8	-2.2	51.0	13.5	even	ok

Figure S19. HRESIMS spectrum of cyclocitrinoic acid B (**2**).

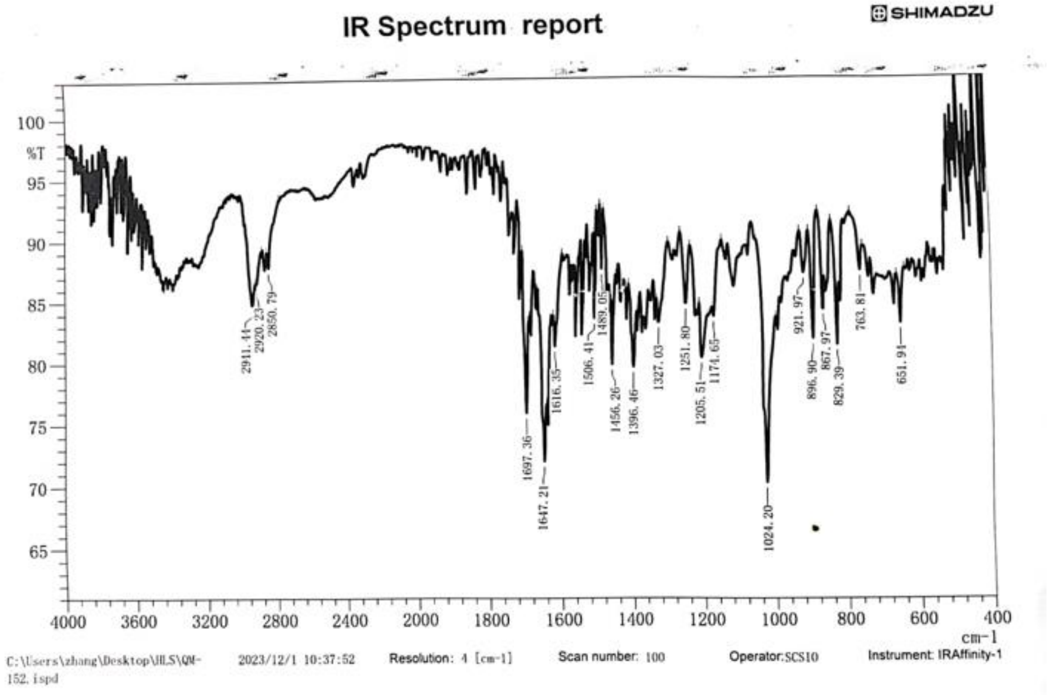

Figure S20. IR spectrum of cyclocitrinoic acid B (**2**).

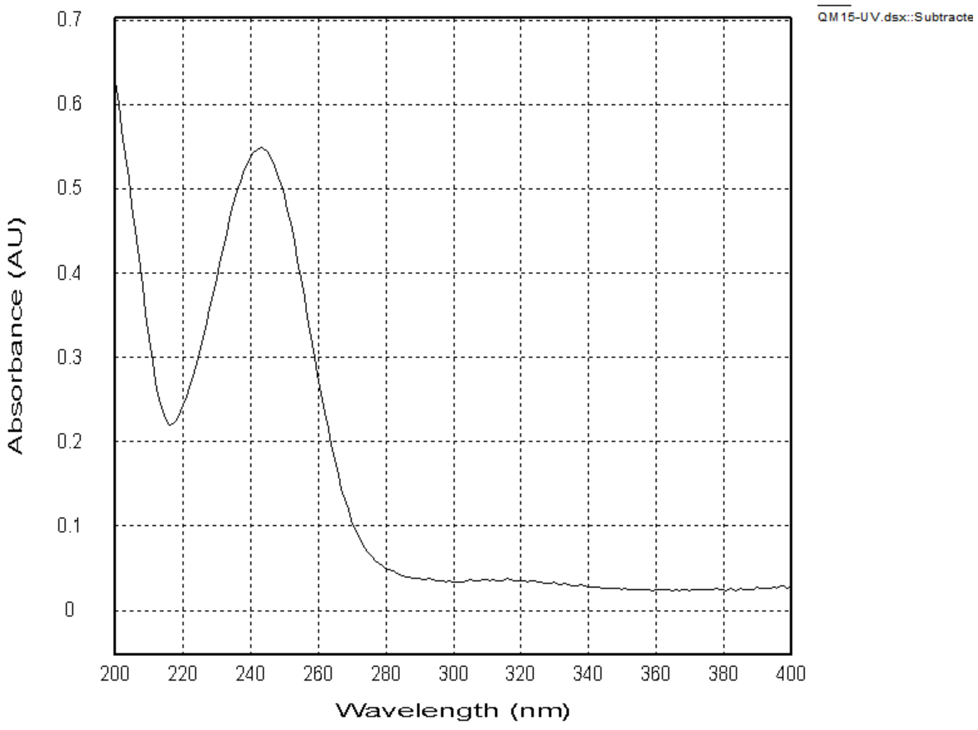

Figure S21. UV spectrum of cyclocitrinoic acid B (**2**) in MeOH.

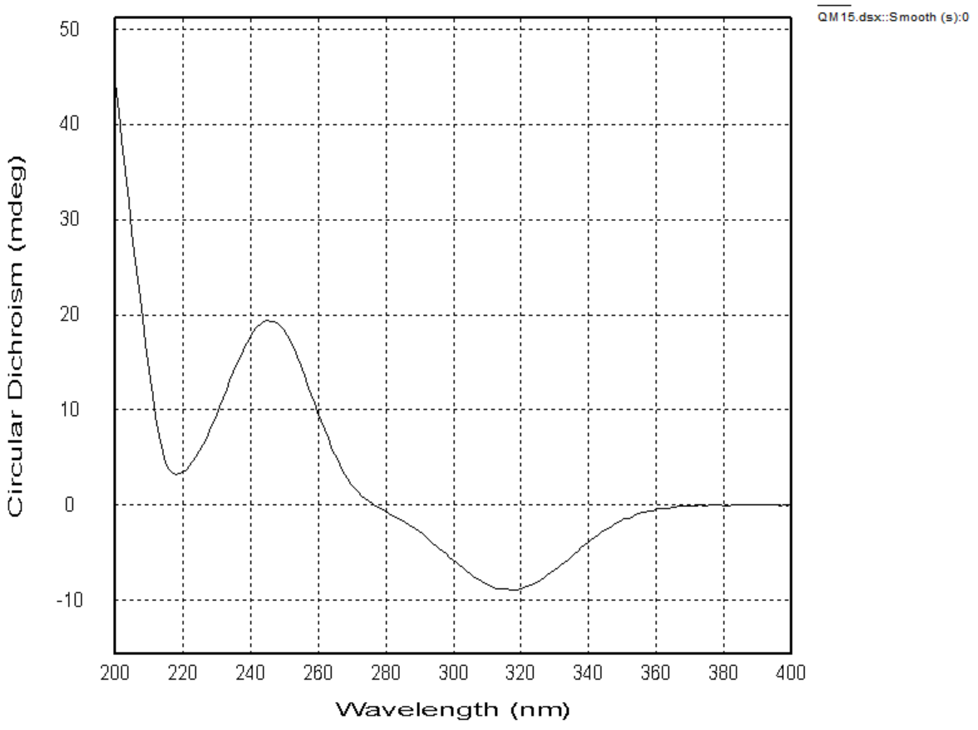

Figure S22. ECD spectrum of cyclocitrinoic acid B (**2**) in MeOH.

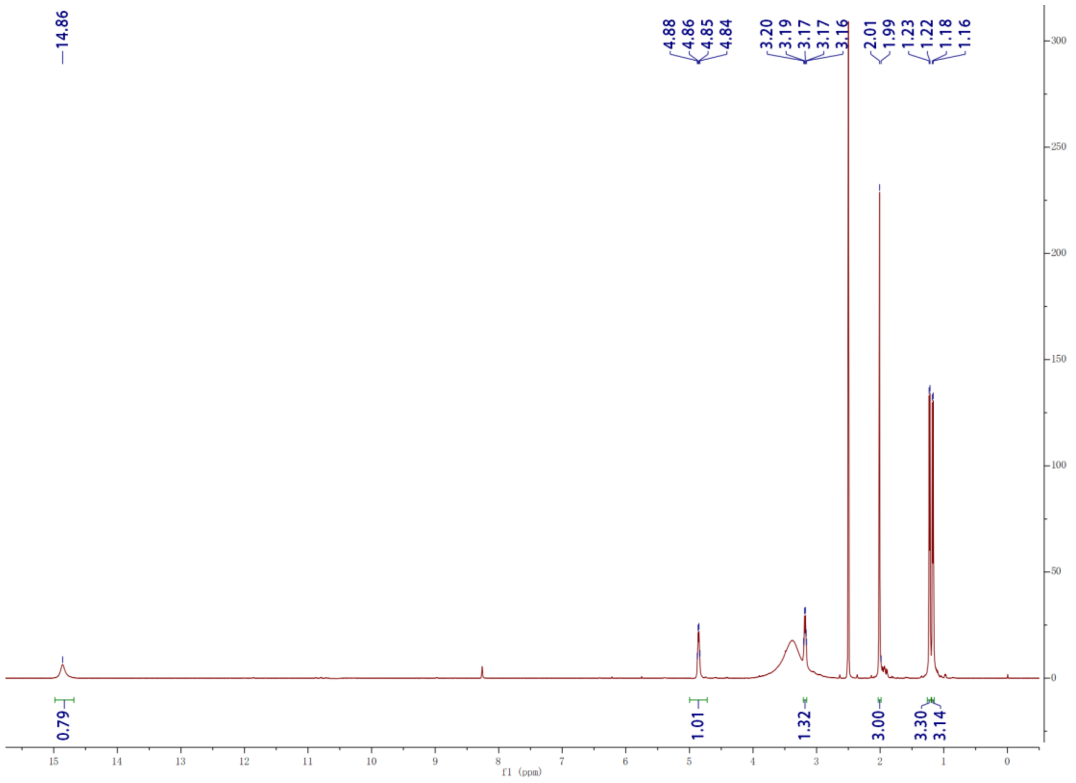

Figure S23. ^{1}H NMR spectrum of (3*R*,4*S*)-6,8-dihydroxy-3,4,5-trimethyl-7-carboxamidelisocoumarin (**10**) in DMSO-d_6.

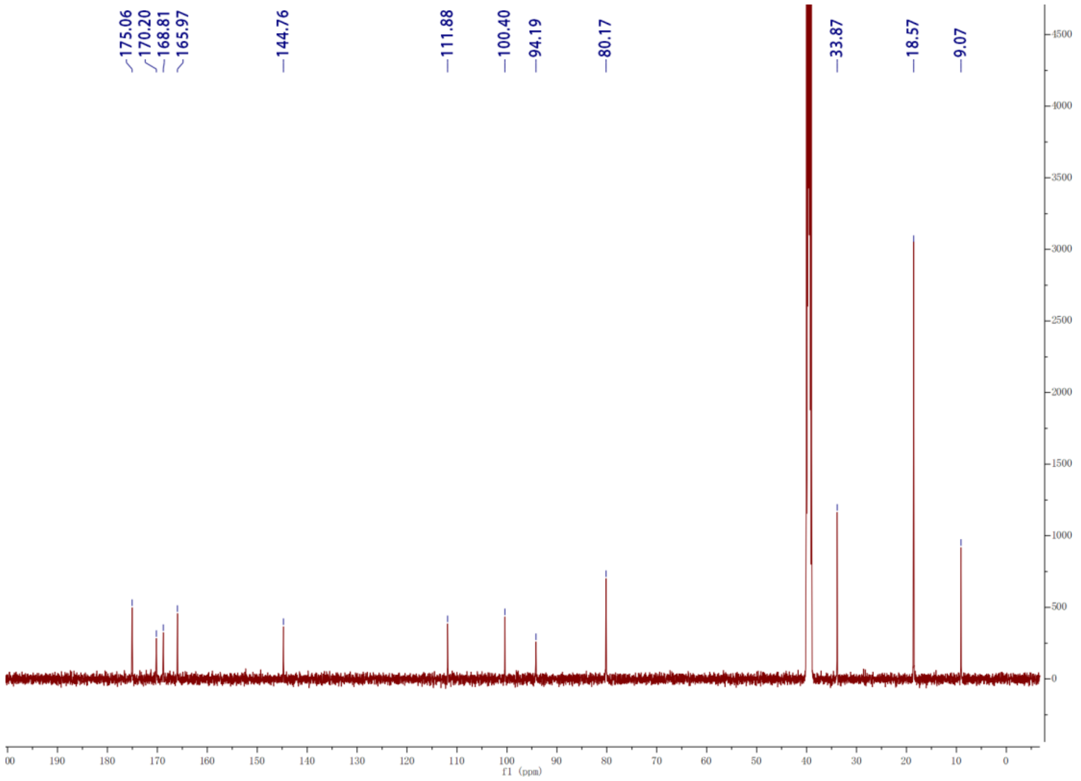

Figure S24. ^{13}C NMR spectrum of (3*R*,4*S*)-6,8-dihydroxy-3,4,5-trimethyl-7-carboxamidelisocoumari (**10**) in DMSO-d_6.

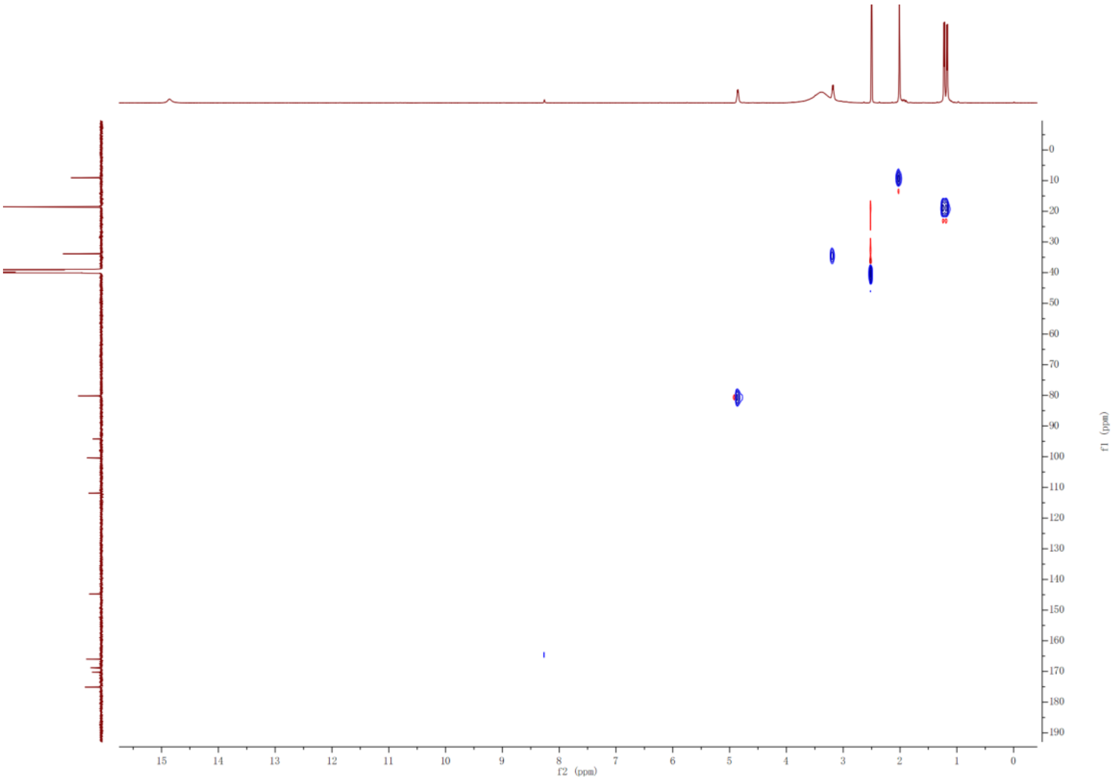

Figure S25. HSQC spectrum of (3*R*,4*S*)-6,8-dihydroxy-3,4,5-trimethyl-7-carboxamidelisocoumari (**10**) in DMSO-d_6.

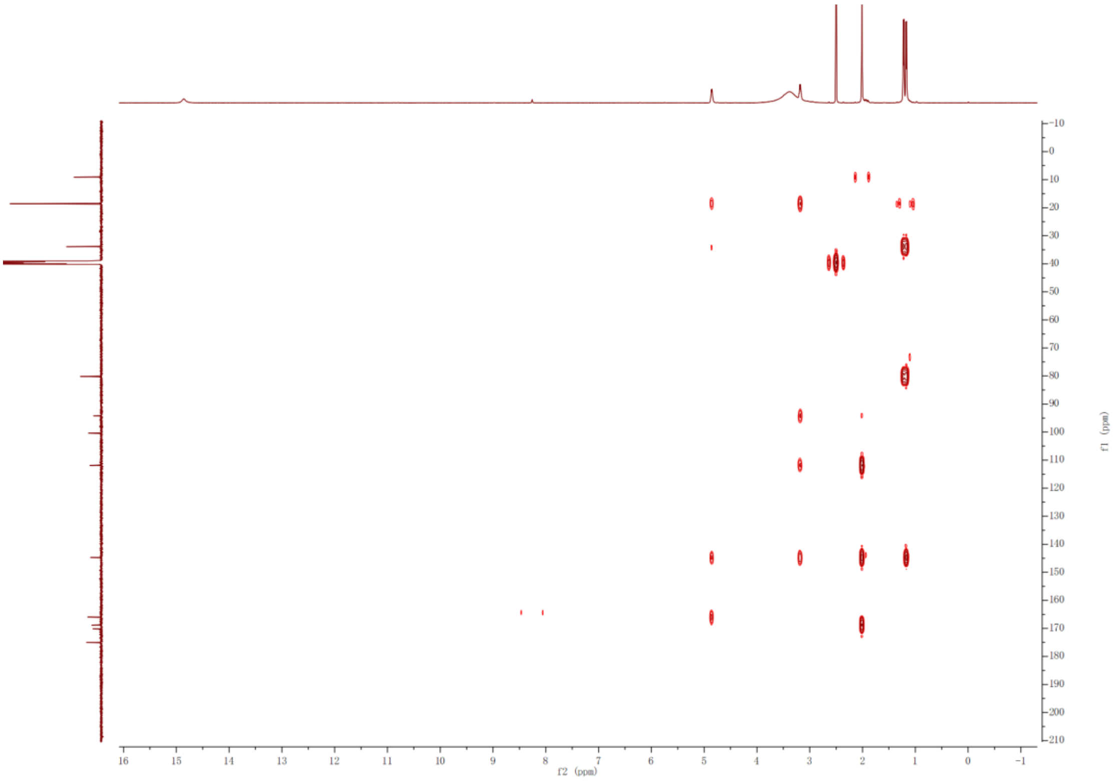

Figure S26. HMBC spectrum of (3*R*,4*S*)-6,8-dihydroxy-3,4,5-trimethyl-7-carboxamidelisocoumari (**10**) in DMSO-d_6.

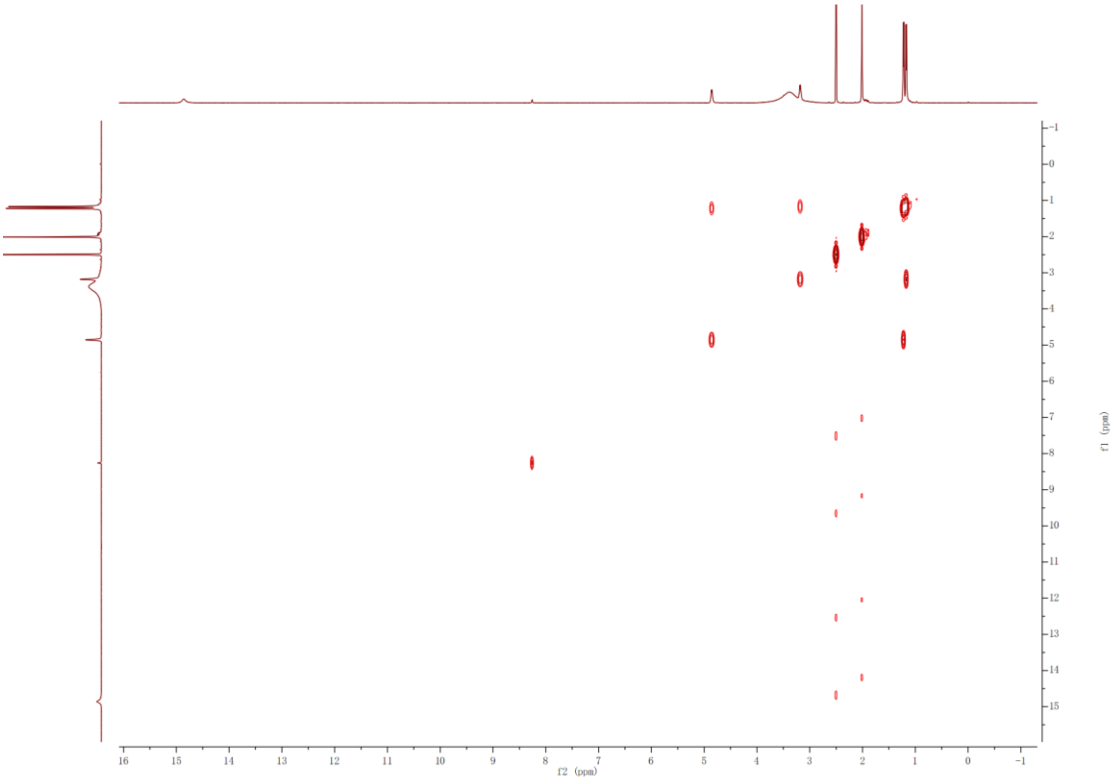

Figure S27. ¹H-¹H COSY spectrum of (3*R*,4*S*)-6,8-dihydroxy-3,4,5-trimethyl-7-carboxamidelisocoumari (**10**) in DMSO-d_6.

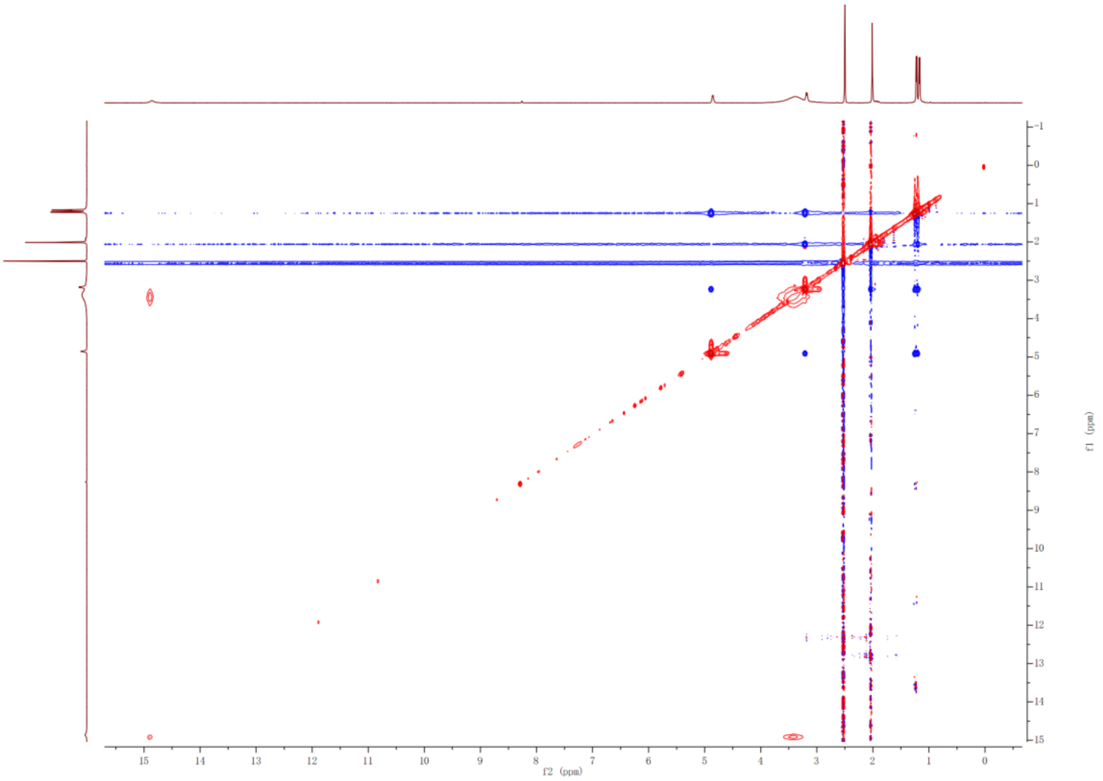

Figure S28. NOESY spectrum of (3*R*,4*S*)-6,8-dihydroxy-3,4,5-trimethyl-7-carboxamidelisocoumari (**10**) in DMSO-d_6.

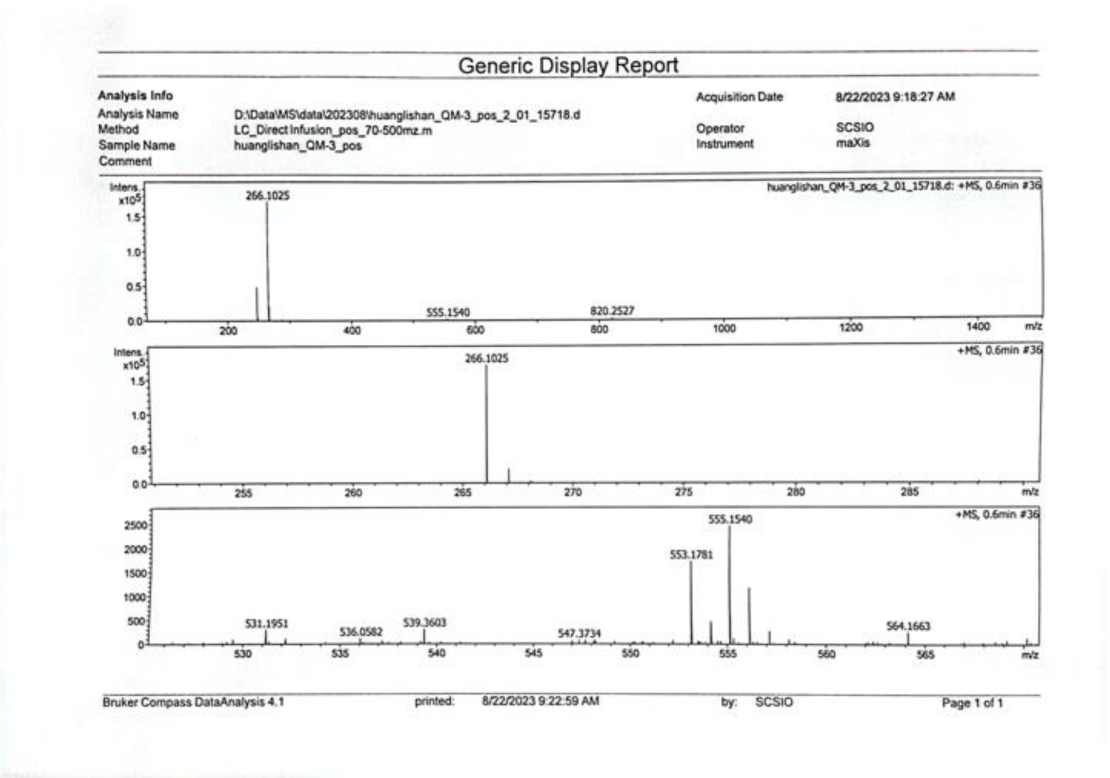

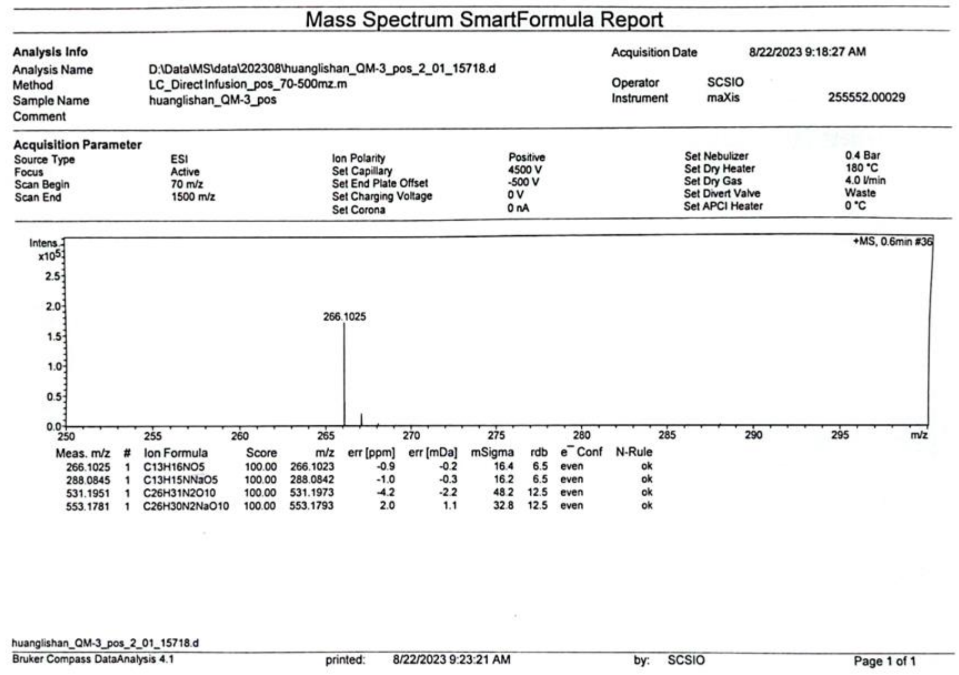

Figure S29. HRESIMS spectrum of (3*R*,4*S*)-6,8-dihydroxy-3,4,5-trimethyl-7-carboxamidelisocoumari (**10**).

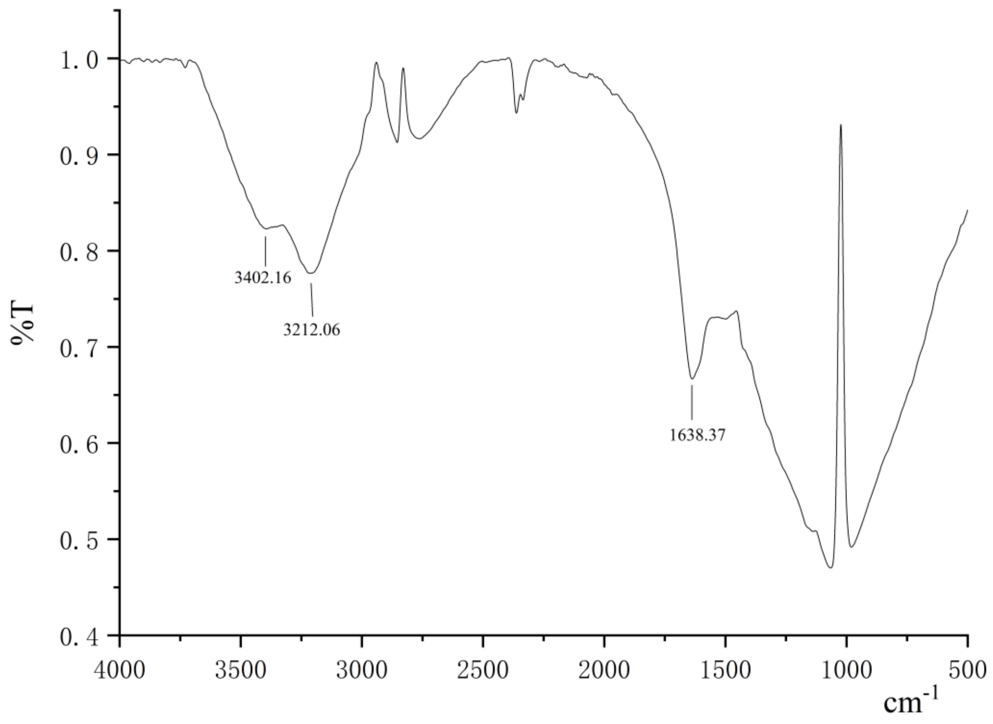

Figure S30. IR spectrum of (3*R*,4*S*)-6,8-dihydroxy-3,4,5-trimethyl-7-carboxamidelisocoumari (**10**).

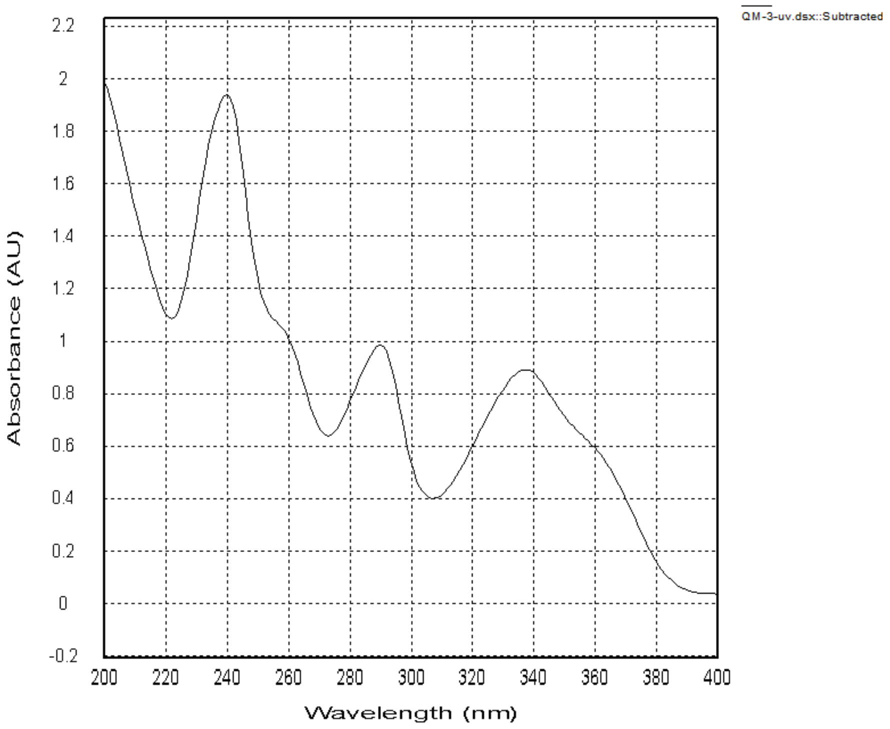

Figure S31. UV spectrum of (3*R*,4*S*)-6,8-dihydroxy-3,4,5-trimethyl-7-carboxamidelisocoumari (**10**) in MeOH.

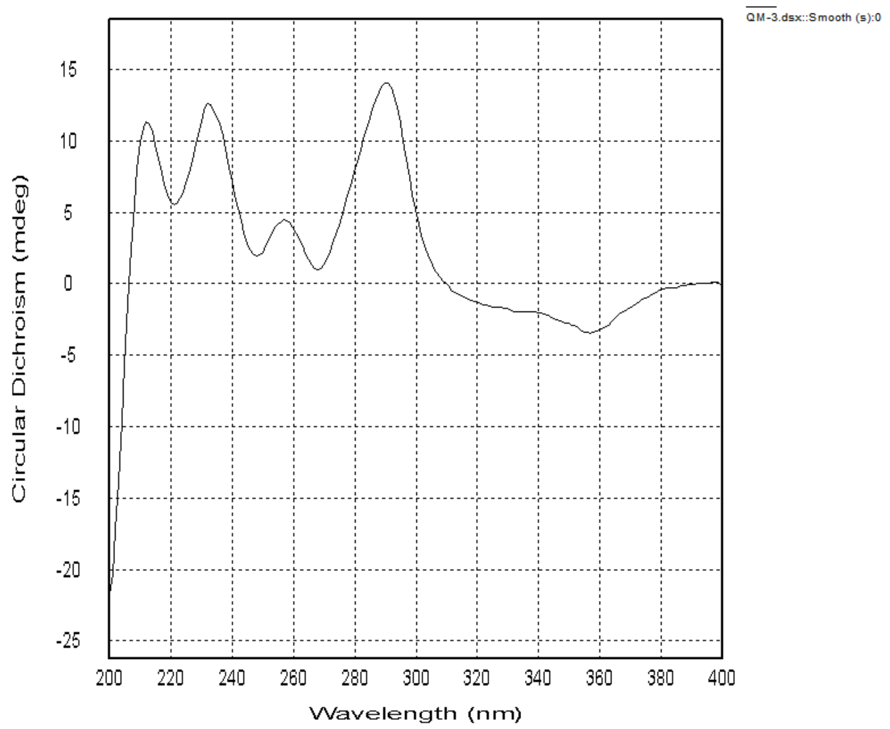

Figure S32. ECD spectrum of (3*R*,4*S*)-6,8-dihydroxy-3,4,5-trimethyl-7-carboxamidelisocoumari (**10**) in MeOH.

The strain's (*Penicillium* sp. SCSIO 41429) ITS 1 and 4 sequence of the rDNA.

TGATATGCTTAAGTTCAGCGGGTATCCCTACCTGATCCGAGGTCAACCTGAGATAATTA
AAGGTTGGGGGTCGGCTGGCGCCGGCCGGGCCTACTAGAGCGGGTGACGAAGCCCCA
TACGCTCGAGGACCGGACGCGGTGCCGCCGCTGCCTTTCGGGCCCGTCCCCCCGGCGG
GGGGGACGGGGCCCAACACACAAGCCGGGCTTGAGGGCAGCAATGACGCTCGGACA
GGCATGCCCTCCGGAATACCAGAGGGCGCAATGTGCGTTCAAAGACTCGATGATTCAC
TGAATTCTGCAATTCACATTAGTTATCGCATTTCGCTGCGTTCTTCATCGATGCCGGAAC
CAAGAGATCCGTTGTTGAAAGTTTTAACTAATTTCGTTATAGGTCTCAGACTGCAACTT
CAGACAGCGTTCAGGGGGGCCGTCGGCGGGCGCGGGGCCCGCCGAGGCAACATAGGT
TCGGGCAACACGGGTGGGAGGTTGGGCCCCGAGGGGCCCGCACTCGGTAATGATCCT
TCCGCAGGTTCACCTACGGAAACCTTGTTACGACTTTTACTTCCT

Table S1. Energies at **1** B3LYP/6-311G(d,p) level in methanol.

Configuration	Conformer	E (Hartree)	E (kcal/mol)	Population (%)
A1-**1**	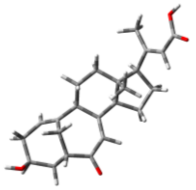	−2560.03016795	−1606442.99467484	0.90%
A2-**1**	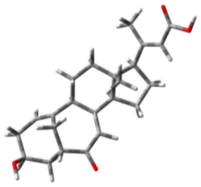	−2562.15290958	−1607775.03500043	37.96%
A3-**1**	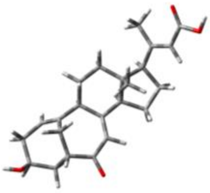	−2562.14420432	−1607769.57236532	18.57%
A4-**1**	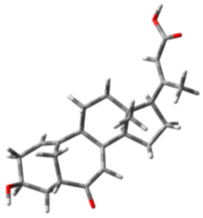	−2561.51813792	−1607376.70981222	24.81%
A5-**1**	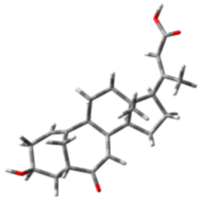	−2561.47181494	−1607347.64170978	12.75%

A6-**1**	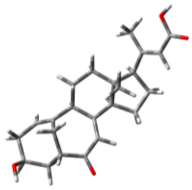	−2563.05378923	−1608340.34544964	3.13%
A7-**1**	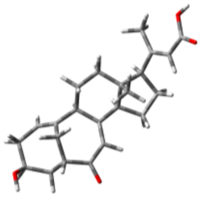	−2563.10830705	−1608374.55589453	18.79%
B1-**1**	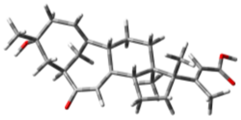	−2560.03016795	−1606442.99467484	0.90%
B2-**1**	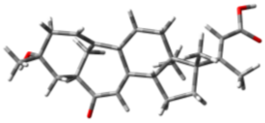	−2562.15290958	−1607775.03500043	37.96%
B3-**1**	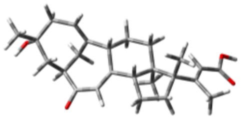	−2562.14420432	−1607769.57236532	18.57%
B4-**1**	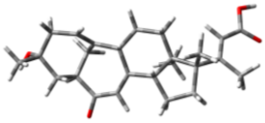	−2561.51813792	−1607376.70981222	24.81%
B5-**1**	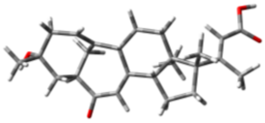	−2561.47181494	−1607347.64170978	12.75%
B6-**1**	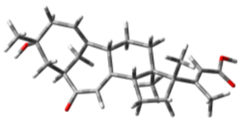	−2563.05378923	−1608340.34544964	3.13%
B7-**1**	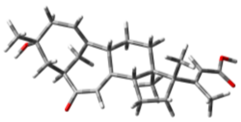	−2563.10830705	−1608374.55589453	18.79%

Table S2. Energies at **2** PCM/mPW1PW91/6-311+G(d,p) level in dimethylsulfoxide.

Configuration	Conformer	E (Hartree)	E (kcal/mol)	Population (%)
A1-**2**	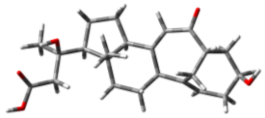	−2803.39066749	−1759153.99572419	67.15%
A2-**2**	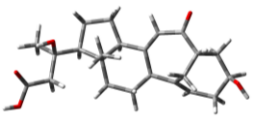	−2803.00628750	−1758912.79366504	28.59%
A3-**2**	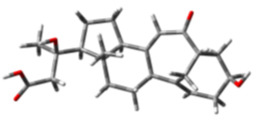	−2804.00146696	−1759537.27812824	4.26%
B1-**2**	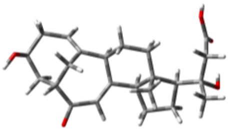	−2837.41134876	−1780502.29301634	5.20%
B2-**2**	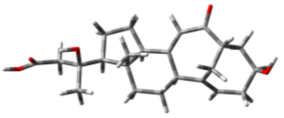	−2789.00345037	−1750125.88173873	66.81%
B3-**2**	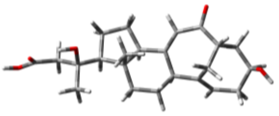	−2788.67221884	−1749918.03083807	27.69%
B4-**2**	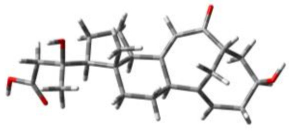	−2811.93863025	−1764517.92270236	0.06%
B5-**2**	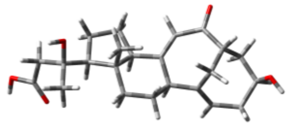	−2811.80805135	−1764435.98321630	0.24%

Table S3. Energies at **10** B3LYP/6-311G(d,p) level in methanol.

Configuration	Conformer	E (Hartree)	E (kcal/mol)	Population (%)
A-(3R, 4*S*)-**10**	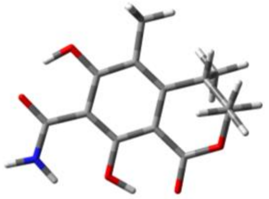	−1528.96302929	−959438.67313195	100.00%

B-(3*S*, 4*R*)-**10**	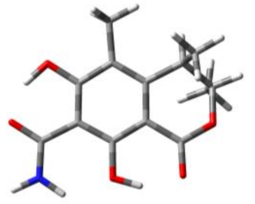	−1528.92131701	−959412.49828415	100.00%
